# Prognostic value and immune landscapes of immunogenic cell death-related lncRNAs in hepatocellular carcinoma

**DOI:** 10.1042/BSR20230634

**Published:** 2023-09-05

**Authors:** Wanying Chen, Kexin Shu, Chenxi Cai, Jiatong Ding, Xin Zhang, Wenxiong Zhang, Kang Wang

**Affiliations:** 1 Department of Thoracic Surgery, The Second Affiliated Hospital of Nanchang University, Nanchang 330006, China; 2 Jiangxi Medical College, Nanchang University, Nanchang 330006, China; 3 Department of Hepatobiliary Surgery, The Second Affiliated Hospital of Nanchang University, Nanchang 330006, China; 4 Department of Traditional Chinese Medicine, The Second Affiliated Hospital of Nanchang University, Nanchang 330006, China

**Keywords:** Anticancer drug sensitivity, Hepatocellular carcinoma, Immunogenic cell death, Long non-coding RNA, Prognostic signature

## Abstract

Background: Both immunogenic cell death (ICD) and long noncoding RNAs (lncRNAs) are strongly associated with tumor development, but the mechanism of action of ICD-associated lncRNAs in hepatocellular carcinoma (HCC) remains unclear.

Methods: We collected data from 365 HCC patients from The Cancer Genome Atlas (TCGA) database. We formulated a prognostic signature of ICD-associated lncRNAs and a nomogram to predict prognosis. To explore the potential mechanisms and provide clinical guidance, survival analysis, enrichment analysis, tumor microenvironment analysis, tumor mutation burden (TMB), and drug sensitivity prediction were conducted based on the subgroups obtained from the risk score.

Results: A prognostic signature of seven ICD-associated lncRNAs was constructed. Kaplan–Meier (K-M) survival curves showed a more unfavorable outcome in high-risk patients. The nomogram had a higher predictive value than the nomogram constructed without the risk model. Enrichment analysis confirmed that risk lncRNAs were closely associated with cell proliferation and mitosis. Most of the immune checkpoints currently used in therapy (e.g., PDCD1 and CTLA4) appeared to be elevated in high-risk patients. Tumor microenvironment analysis showed differential expression of lymphocytes (including natural killer cells, regulatory T cells, etc.) in the high-risk group. TMB had a higher incidence of mutations in the high-risk group (*P*=0.004). Chemotherapy drug sensitivity prediction provides effective guidelines for individual therapy. RT-qPCR of human HCC tissues verified the accuracy of the model.

Conclusion: We constructed an effective prognostic signature for patients with HCC using seven ICD-lncRNAs, which provides guidance for the prognostic assessment and personalized treatment of patients.

## Introduction

Hepatocellular carcinoma is the sixth most prevalent primary carcinoma and the third most common cause of cancer-related death globally. Hepatocellular carcinoma (HCC) constitutes 90% of liver cancer cases and is one of the most prevalent malignancies with rapid progression and deterioration and an adverse prognosis, with a 5-year survival rate of <50% [1]. Tumor node metastasis classification (TMN) staging is one of the current methods for assessing the prognosis of HCC [2], but its ability to estimate the longer-range prognosis of patients is diminished because it does not account for factors such as cirrhosis [3].

Immunogenic cell death (ICD), a form of apoptosis mediated by activation of immune cells through specific antigen recognition [4], is closely affiliated with tumor development and extensively used in the treatment of tumors (including HCC) [5,6]. In addition, the combination of long noncoding RNAs (lncRNAs) has shown good results as a novel prognostic signature and clinical guidance for patients with cancer [7,8]. ICD-associated lncRNAs have been used in the prognostic assessment of head and neck squamous cell carcinoma, uveal melanoma, low-grade glioma, stomach adenocarcinoma and osteosarcoma [9–13]. The established prognostic signatures demonstrated high predictive accuracy, indicating the importance of ICD-related lncRNAs in the construction of tumor prognostic signatures. Therefore, a novel combination of biomarkers for ICD is needed for the effective prediction of HCC. In the present study, we associated ICD and HCC with lncRNAs to develop a predictive model that is valid and accurate at an early stage, applicable to all stages of HCC and able to provide guidance for personalized treatment after evaluation.

## Materials and methods

### Collection and disposing of HCC-related data and ICD genetic data

**Figure 1:**
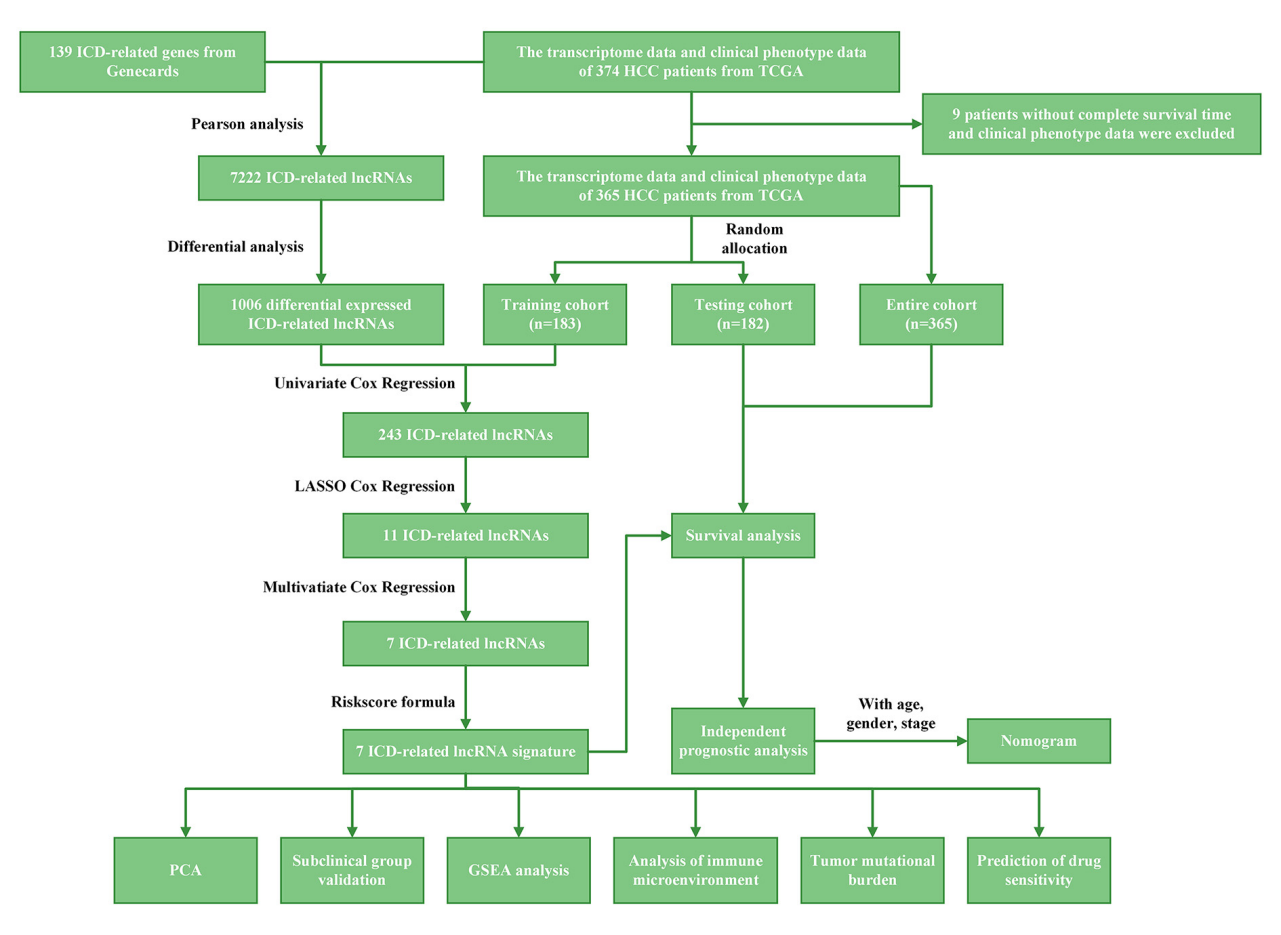
Flow diagram of the study design

We obtained transcriptomic gene expression information, single-nucleotide mutation information, survival information, and clinical phenotype data from The Cancer Genome Atlas (TCGA) database (https://portal.gdc.cancer.gov/, accessed on October 30, 2022) for patients with HCC, yielding a series of 424 specimens, including 374 tumor tissue specimens and 50 normal tissue specimens. The data were processed as shown in Figure 1. Nine tumor tissue specimens with no survival information were excluded. The training cohort and test cohort were obtained after random grouping, and the patient-specific information is shown in Table 1.

**Table 1:** Clinical information of 365 HCC patients from TCGA database

Feature	Train cohort (*n*=183)		Test cohort (*n*=182)		Entire cohort (*n*=365)	
	*n*	%	*n*	%	*n*	%
**Age**						
<65	101	55.19	115	63.19	216	59.18
≥65	82	44.81	67	36.81	149	40.82
**Status**						
Alive	123	67.21	111	60.99	234	64.11
Dead	60	32.79	71	39.01	131	35.89
**Gender**						
Female	61	33.33	58	31.87	119	32.60
Male	122	66.67	124	68.13	246	67.40
**Stage**						
Stage I	86	46.99	84	46.15	170	46.58
Stage II	42	22.95	42	23.08	84	23.01
Stage III	38	20.77	45	24.73	83	22.74
Stage IV	2	1.09	2	1.09	4	1.10
Unknown	15	8.20	9	4.95	24	6.57
**T stage**						
T1	91	49.73	89	48.90	180	49.32
T2	44	24.04	47	25.82	91	24.93
T3	40	21.86	38	20.88	78	21.37
T4	6	3.28	7	3.85	13	3.56
Unknown	2	1.09	1	0.55	3	0.82
**N stage**						
N0	122	66.67	126	69.23	248	67.95
N1	1	0.55	3	1.65	4	1.09
N2	0	0	0	0	0	0
N3	0	0	0	0	0	0
Unknown	60	32.78	53	29.12	113	30.96
**M stage**						
M0	129	70.49	134	73.63	263	72.06
M1	2	1.09	1	0.55	3	0.82
Unknown	52	28.42	47	25.82	99	27.12

Abbreviations: HCC, hepatocellular carcinoma; TCGA, The Cancer Genome Atlas.

A total of 139 ICD-associated genes were obtained from Genecards (https://www.genecards.org/, accessed on October 30, 2022), according to a score>35’ [14]. Pearson correlation analysis (based on |*R*|^2^>0.3, *P*<0.001) was performed by the corr.test function in R (4.2.1).

### Screening for ICD-associated DELs

To identify differentially expressed genes between tumor specimens and normal specimens, we used the ‘DESeq2’ package in R (4.2.1) to conduct differential analysis of lncRNAs obtained from Pearson correlation analysis (|log_2_Fold Change|>1 and *P*<0.05) [15].

### Formulation of the prognostic signature

Univariate Cox, LASSO regression, and multivariate Cox analyses were performed sequentially in the training cohort by means of the ‘survival’ package in R (4.2.1) [16]. The following formula was used to calculate the risk score for each segment: risk score = (coefficient1 * gene1 expression) +… + (coefficientN * geneN expression). Patients were divided into subgroups according to risk scores; patients with scores above the median were assigned to the high-risk group, and the remaining patients were assigned to the low-risk group.

### Validation of the prognostic signature

For the prognostic signature, we performed survival analysis for the training cohort, test cohort, and entire cohort using the ‘survival’ package in R (4.2.1). Time-dependent ROC curves were plotted by using the ‘rms’ package and time-ROC package [17].

To validate the prognostic signature in relation to other clinical information, we used the ‘survival’ package for independent prognostic analysis and the ‘ggDCA’ package, the ‘rms’ package, and the ’foreign’ package for DCA [18].

In accordance with the different clinical characteristics, we calculated the risk scores in different clinical subgroups and grouped them for survival analysis to visualize the applicability of our risk model.

### Construction of the nomogram and calibration curves

To yield a more comprehensive and accurate risk prediction model, we combined the clinical information of HCC patients and plotted a nomogram and calibration curves using the ‘survival’ package, ‘regplot’ package, and ‘rms’ package [19].

### PCA

To better visualize the divergent genetic performance among high- and low-risk patients, we performed PCA of complete patient genes, ICD-associated genes, ICD-lncRNA genes, and risk-ICD-lncRNA genes using the ‘scatterplot3d’ package in R [20].

### Pathway enrichment analysis

We performed pathway enrichment by GSEA (4.3.2) using the groupings obtained by risk score; specifically, the datasets used were biocarta, kegg, pid, reactome, and go in MSigDB [21]. The pathway enrichment analysis criteria were false discovery rate (FDR) < 0.25 and *q*-value < 0.01.

### Tumor microenvironment analysis

We performed immune infiltration analysis of a full set of HCC patients using the CIBERSORT algorithm in R (4.2.1). By using the ‘GSVA’, ‘GSWABase’, and ‘limma’ packages, we performed immune function analysis in both patient groups. We also performed immune scoring using different algorithms (TIMER, CIBERSORT, CIBERSORT-ABS, QUANTISEQ, MCPCOUNTER, XCELL, EPIC) through the timer2.0 website (http://timer.cistrome.org/, accessed on November 4, 2022) [22].

### Immune checkpoint expression

From previous papers, we collected information on immune checkpoints and performed an analysis of diverse checkpoint expression in high- and low-risk patients with the ‘reshape2’ package.

### Tumor mutation burden analysis

By utilizing the ‘maftools’ package in R (4.2.1), we generated waterfall plots of mutation proportions and types and performed analytical calculations of tumor mutation compliance for each of the two patient subgroups according to risk score [23].

### Prediction of chemotherapy drug sensitivity

We downloaded information on the effectiveness of chemotherapy from the GDSC website (https://www.cancerrxgene.org/, accessed on November 4, 2022) and used the ‘oncoPredict’ package to compare the IC50 differences among 198 chemotherapeutic drugs in combination with information from HCC patients [24].

### RT-qPCR of human HCC tissue

Eighty-two surgically resected specimens of HCC patients from the Second Affiliated Hospital of Nanchang University were collected. The excised specimens were placed in storage at −80°C after rapid freezing by liquid nitrogen prior to the experiment. We grouped patients and validated the accuracy of the risk gene signature for determining prognosis. Permission for the investigation was granted by the Ethics Committee of the Second Affiliated Hospital of Nanchang University, and informed consent was sought from all patients.

RNA was reverse transcribed at 45°C for 1 h using the SureScript First-Strand cDNA Synthesis kit (GeneCopoeia, China). BlazeTaq SYBR Green qPCR master mix (GeneCopoeia, China) and Applied Biosystems 7500 Fast Real-Time PCR System (Applied Biosystems) were used for subsequent RT-qPCR analysis. The relevant primers used are shown in Supplementary Table S1. The expression of each gene is expressed as the 2^ΔΔCt^ value. For each specimen, we conducted three experiments to extract the mean value.

To verify the relevance of ICD-associated genes to HCC, we compared the differential expression of ICD-associated genes between HCC and normal tissues through the Human Protein Atlas database (https://www.proteinatlas.org/, accessed on January 25, 2023).

## Results

### Screening for risk ICD-lncRNAs

**Figure 2:**
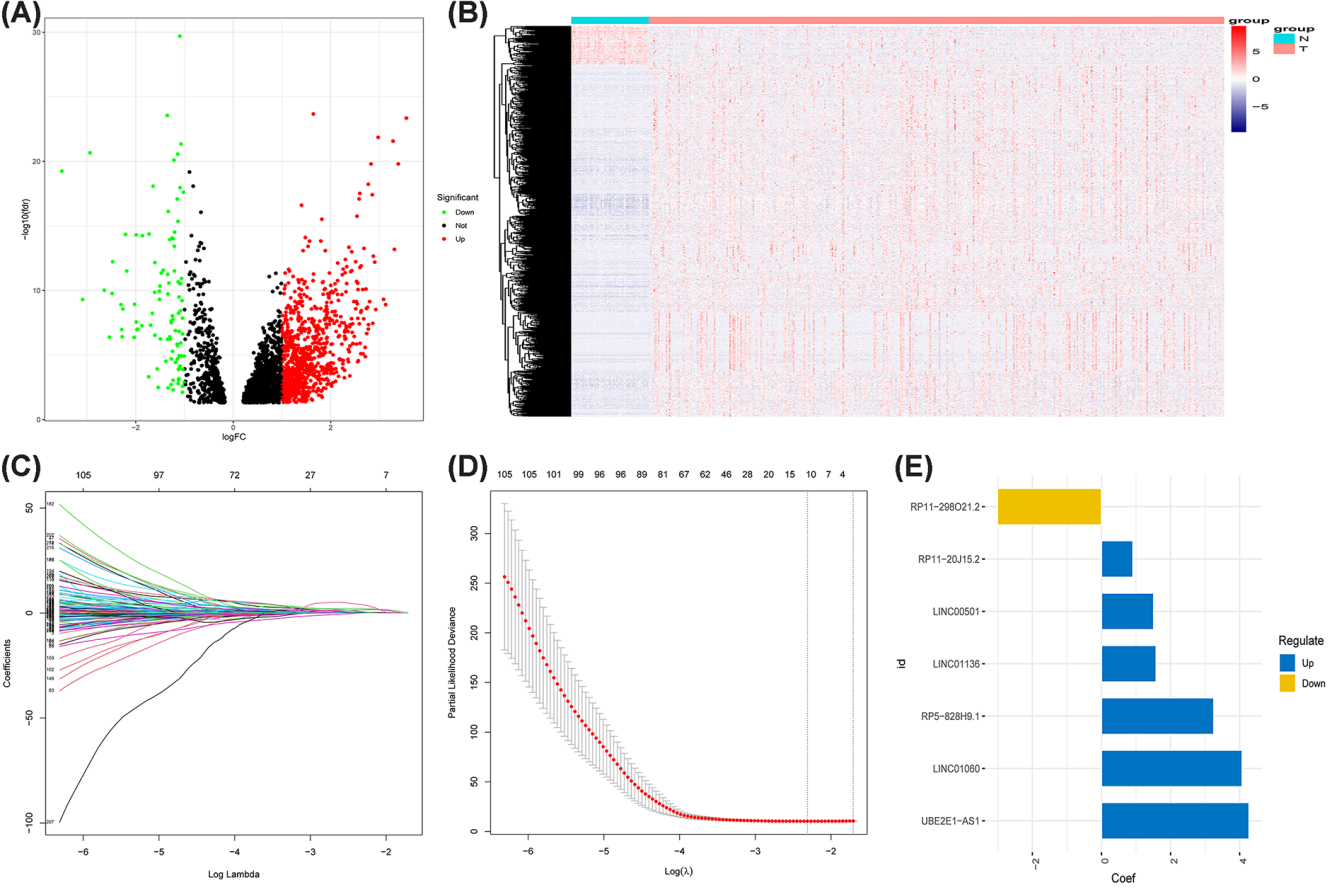
Identification of ICD-associated lncRNAs (**A**) Volcano plot and (**B**) heat map of 1006 differentially expressed ICD-associated lncRNAs. (**C,D**) LASSO regression analysis to determine the most appropriate number of ICD-associated lncRNAs for analysis. (**E**) Deviation plots of seven ICD lncRNAs showing the up- and down-regulated expression of ICD-related lncRNAs used for analysis.

A total of 7222 ICD-lncRNAs were screened out of 14831 lncRNAs by Pearson correlation analysis. Comparing the gene expression levels in 365 tumor tissue specimens and 50 normal tissue specimens, we detected 1006 DELs among 7222 ICD-lncRNAs, of which 906 lncRNAs were up-regulated and 100 were down-regulated (Figure 2A,B).

After univariate Cox analysis of DELs at *P*<0.05, 243 ICD-associated lncRNAs were obtained (Supplementary Table S2). LASSO regression was used to further screen the relevant lncRNAs (Figure 2C,D), and 11 genes for stable construct models were obtained. We performed multivariate Cox regression analysis on the 11 lncRNAs and finally identified 7 lncRNAs, LINC01136, RP11.298O21.2, RP11.20J15.2, LINC00501, UBE2E1.AS1, LINC01060, and RP5.828H9.1 (Supplementary Table S3 and Figure 2E).

### Construction of the prognostic signature

The multivariate Cox analysis results were combined using the following formula: risk score = (1.576275843 × LINC01136 expression) + (−3.014558831 × RP11.298O21.2 expression) + (0.911419424 × RP11.20J15.2 expression) + (1.50737826 × LINC00501 expression) + (4.267512039 × UBE2E1.AS1 expression) + (4.066672785 × LINC01060 expression) + (3.247792567 × RP5.828H9.1 expression). Using this formula, we assigned risk scores to all HCC patients, and we categorized the patients into high- (*n*=182) and low-risk groups (*n*=183). The clinical information of patients in each group is shown in Table 2.

**Table 2:** Clinical information of HCC patients in two risk groups

Feature	High-risk group (*n*=182)		Low-risk group (*n*=183)	
	*n*	%	*n*	%
**Age**				
<65	114	62.64	102	55.74
≥65	68	37.36	81	44.26
**Status**				
Alive	103	56.59	131	71.58
Dead	79	43.41	52	28.42
**Gender**				
Female	63	34.62	56	30.60
Male	119	65.38	127	69.40
**Stage**				
Stage I	70	38.46	100	54.64
Stage II	47	25.83	37	20.22
Stage III	49	26.92	34	18.58
Stage IV	4	2.20	0	0
Unknown	12	6.59	12	6.56
**T stage**				
T1	75	41.21	105	57.38
T2	52	28.57	39	21.31
T3	45	24.73	33	18.03
T4	10	5.49	3	1.64
Unknown	0	0	3	1.64
**N stage**				
N0	127	69.78	121	66.12
N1	4	2.20	0	0
N2	0	0	0	0
N3	0	0	0	0
Unknown	51	28.02	62	33.88
**M stage**				
M0	138	75.82	125	68.31
M1	3	1.65	0	0
Unknown	41	22.53	58	31.69

Abbreviations: HCC, hepatocellular carcinoma; T, Tumor; N, Node; M, Metastasis.

**Figure 3:**
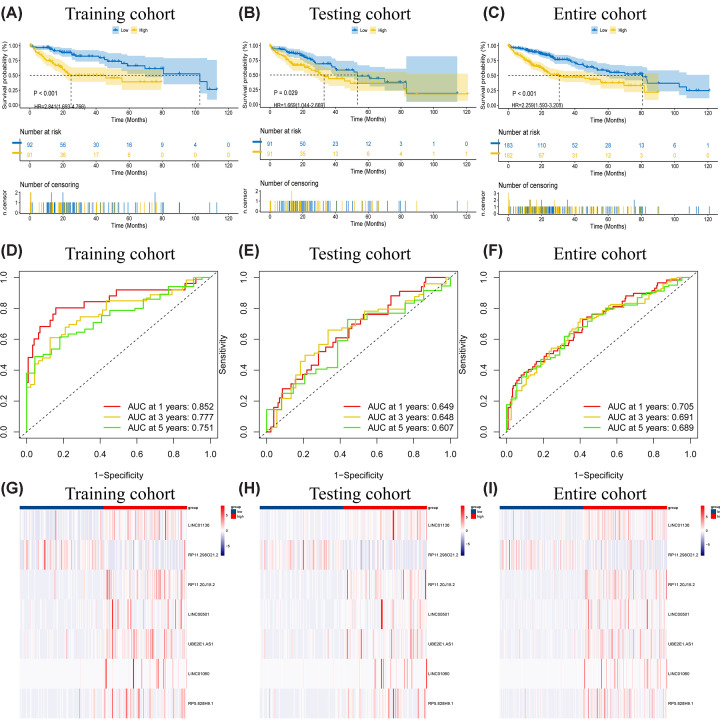
Validation of independent prognostic signatures K-M survival analyses of (**A**) training cohort, (**B**) testing cohort, and (**C**) entire cohort demonstrated significant survival differences between the high and low risk groups distinguished in this prognostic model. The AUC value of (**D**) training cohort, (**E**) testing cohort, and (**F**) entire cohort illustrated the accuracy of the prognostic model in predicting 1-, 3-, and 5-year survival rates. Heatmaps in (**G**) training cohort, (**H**) testing cohort, and (**I**) entire cohort showed that the expression trends of the seven lncRNAs in the three groups were consistent.

Survival analysis was performed for the training cohort, the testing cohort, and the entire cohort (Figure 3A–C), with *P-*values of <0.001, 0.029, and <0.001, respectively. We subsequently confirmed the correctness of the prognostic signature by time-ROC (Figure 3D–F), demonstrating well-predicted results of the model (training: 1-AUC = 0.852, 3-AUC = 0.777, 5-AUC = 0.751; testing: 1-AUC = 0.649, 3-AUC = 0.648, 5-AUC = 0.607; entire: 1-AUC = 0.705, 3-AUC = 0.691, 5-AUC = 0.689). Heatmaps were drawn for each subgroup of risk-lncRNAs (Figure 3G–I), which consistently showed that RP11.298O21.2 was highly prevalent in the low-risk group, while LINC01136, RP11.20J15.2, LINC00501, UBE2E1.AS1, LINC01060, and RP5.828H9.1 were highly prevalent in the high-risk group. Heatmaps combining clinical details suggested that differentially expressed genes were only associated with risk score and staging and were independent of age and sex (Supplementary Figure S1A). We generated scatter plots of survival status by risk group for the training, testing, and entire cohorts, which showed that risk group was correlated with survival status (Supplementary Figure S1B–G).

### Validation of the prognostic signature

**Figure 4:**
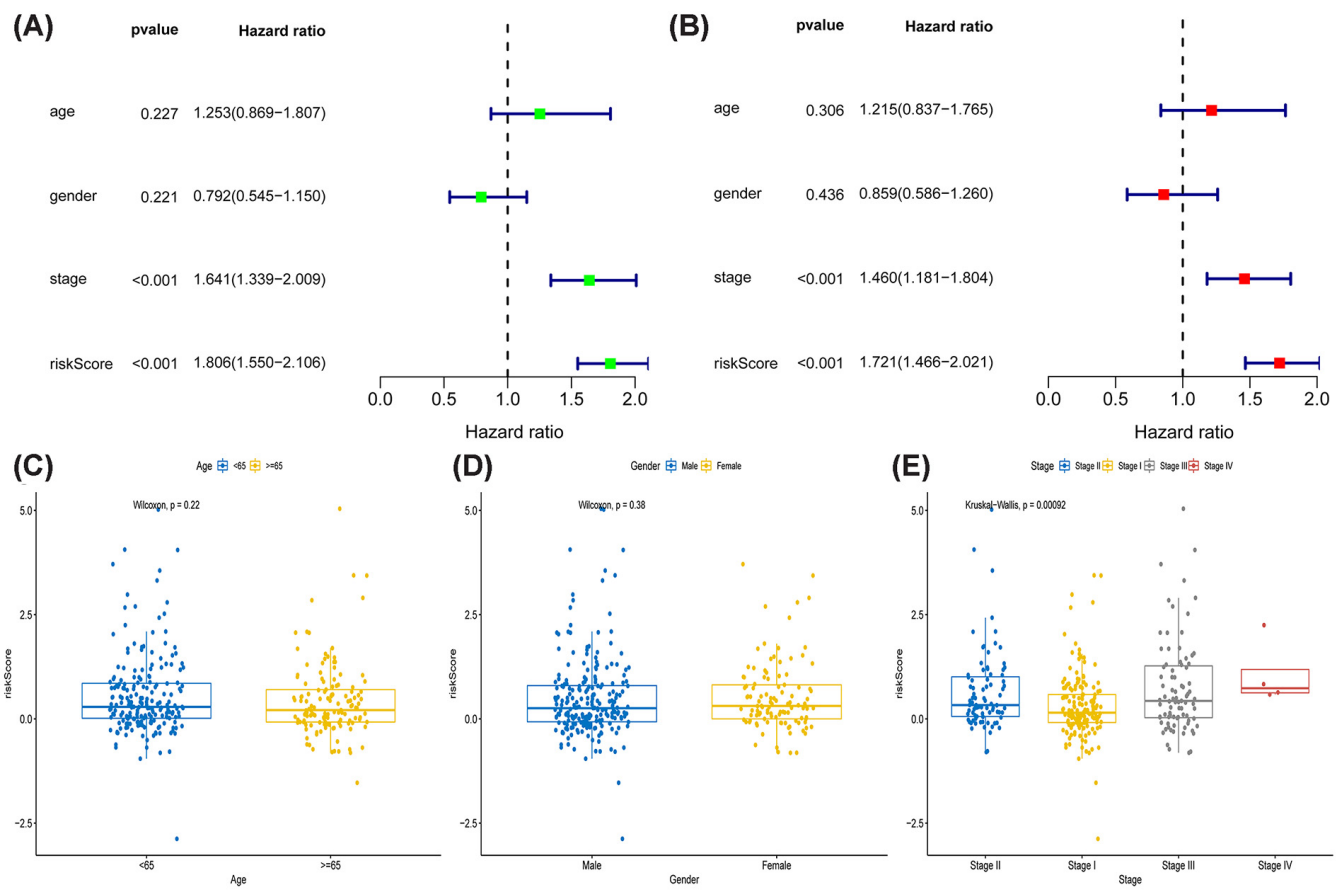
Independent prognostic analysis and clinical correlation analysis (**A**) Independent prognostic univariate analysis and (**B**) independent prognostic multivariate analysis of 4 prognostic correlates illustrated that risk could be an independent prognostic factor for patients with hepatocellular carcinoma. Correlation of risk scores with (**C**) age, (**D**) gender (**E**), and stage.

Patient age, sex, clinical stage and risk score were used as four factors in the analysis for independent prognosis (Figure 4A,B). The results of univariate Cox regression analysis (HR = 1.806, 1.550–2.06, *P*<0.001) and multivariate Cox regression analysis (HR = 1.721, 1.466–2.021, *P*<0.001) indicated that risk score is an individual prognostic factor (Supplementary Table S4). The results of the correlation between the risk score and the three clinical factors indicated that age and sex were not substantially correlated with the risk score, but clinical stage was correlated (Figure 4C–E).

As shown by the survival analysis of different clinical subgroups, the prognostic signature was applicable in all subgroups, especially for patients age <65, female patients, and stage I∼II and T1∼2 patients (Supplementary Figure S2).

PCA was carried out separately for all genes, ICD-associated genes, ICD-associated lncRNAs, and risk-ICD-lncRNAs. 3dPCA plots showed that the seven prognosis-associated lncRNAs were most strikingly different between the high- and low-risk groups (Supplementary Figure S3).

### Nomogram

**Figure 5:**
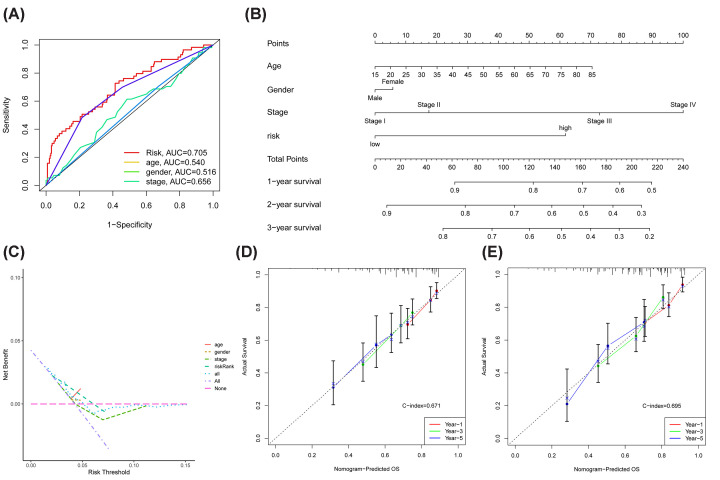
Construction and validation of the nomogram combining four prognostic correlates (**A**) The AUC values of four prognostic correlates demonstrated the accuracy of each correlate in predicting survival. (**B**) A nomogram combining four prognostic correlates (risk, age, gender, and stage) for predicting 1-, 3-, and 5-year survival in patients with hepatocellular carcinoma. (**C**) DCA analysis of four risk factors. Calibration curves for nomogram (**D**) without risk (**E**) and without risk, indicating that the inclusion of the prognostic model helped to improve the accuracy of nomogram in predicting survival.

We plotted ROC curves for the four factors (Figure 5A), and the AUCs were 0.705, 0.540, 0.516, and 0.656 for risk score, age, sex, and clinical stage, respectively. The above results illustrate that the nomogram has good predictive efficacy and provides accurate prognosis evaluation results for HCC patients.

In an attempt to yield a more comprehensive and accurate prognostic score, we integrated the patients’ clinical information with the risk score to derive a nomogram (Figure 5B) to predict the patients’ 1-, 3-, and 5-year survival rates. Decision curve analysis (DCA) further confirmed that the model incorporating the risk score was more worthy of evaluation (Figure 5C). The calibration curve demonstrated the predictive capability of the nomogram (Figure 5D,E), suggesting that the comprehensive nomogram with the risk score (C-index = 0.695) is superior to the nomogram without the risk score (C-index = 0.671). Furthermore, we arbitrarily chose one patient to confirm the feasibility of prediction with the nomogram (Supplementary Figure S4).

### Functional analysis of prognosis-related lncRNAs

**Figure 6:**
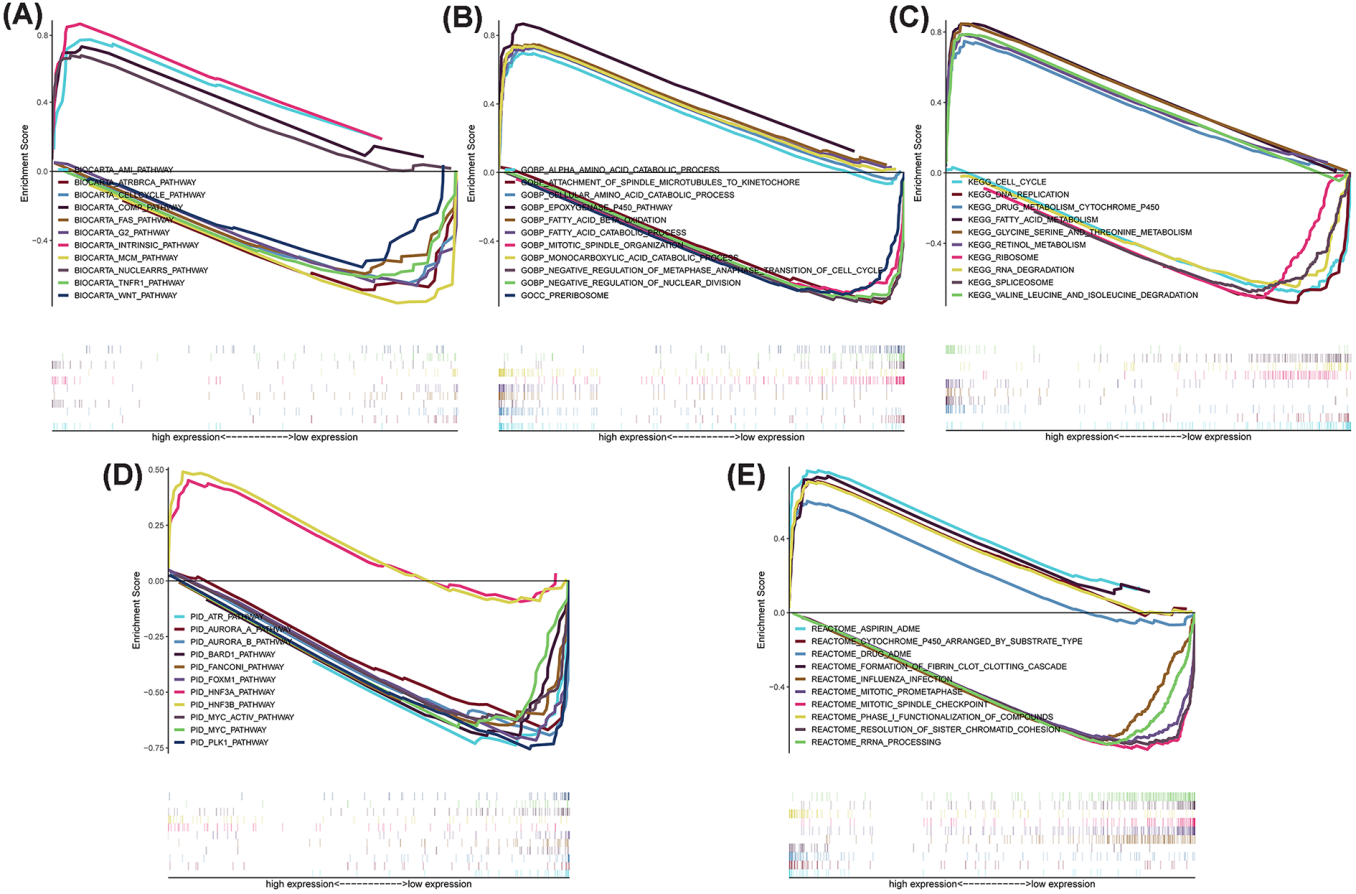
The function of pathways associated with the predicted signatures, as determined via GSEA Enrichment analyses of (**A**) Biocarta, (**B**) GO, (**C**) KEGG, (**D**) Pid, and (**E**) Reactome.

In the Biocarta enrichment analysis, the low-risk gene set was functionally associated with endogenous thrombospondin activation, lipid metabolism and toxic nuclear receptors, and complement pathways; the high-risk genes were relevant to cell cycle regulation, BRCA1/2, and ATR susceptibility in cancer (Figure 6A). In the remaining four gene sets, the pathways in the low-risk group were closely affiliated with substance or drug metabolism, while the high-risk group was chiefly connected with mitosis (Figure 6B–E). The outputs of the GSEA for the high- and low-risk groups are exhibited in Supplementary Tables S5 and S6.

### HCC immune microenvironment

In the immunoinfiltration analysis, antigen-presenting cells (APCs), macrophages, follicular regulatory T lymphocytes (Tfhs), T helper 2 cells (Th2), and regulatory T lymphocytes (Tregs) were highly frequent in the high-risk group; B cells and natural killer (NK) cells were highly expressed in the low-risk group (Supplementary Figure S5A), as detailed in Supplementary Figure S6A–J.

In the immune function analysis, APC_co_stimulation, HLA, and MHC_class_I scores were higher in the high-risk group, while Type_II_IFN_Response scores were higher in the low-risk group (Supplementary Figure S5B).

The immune differential analysis by seven different algorithms is shown in the significance bubble plot, which had similar results to those of ssGSEA, indicating that immune cells tend to infiltrate more in high-risk patients (Supplementary Figure S5C).

### Differential tumor mutation burden in high- and low-risk patients

**Figure 7:**
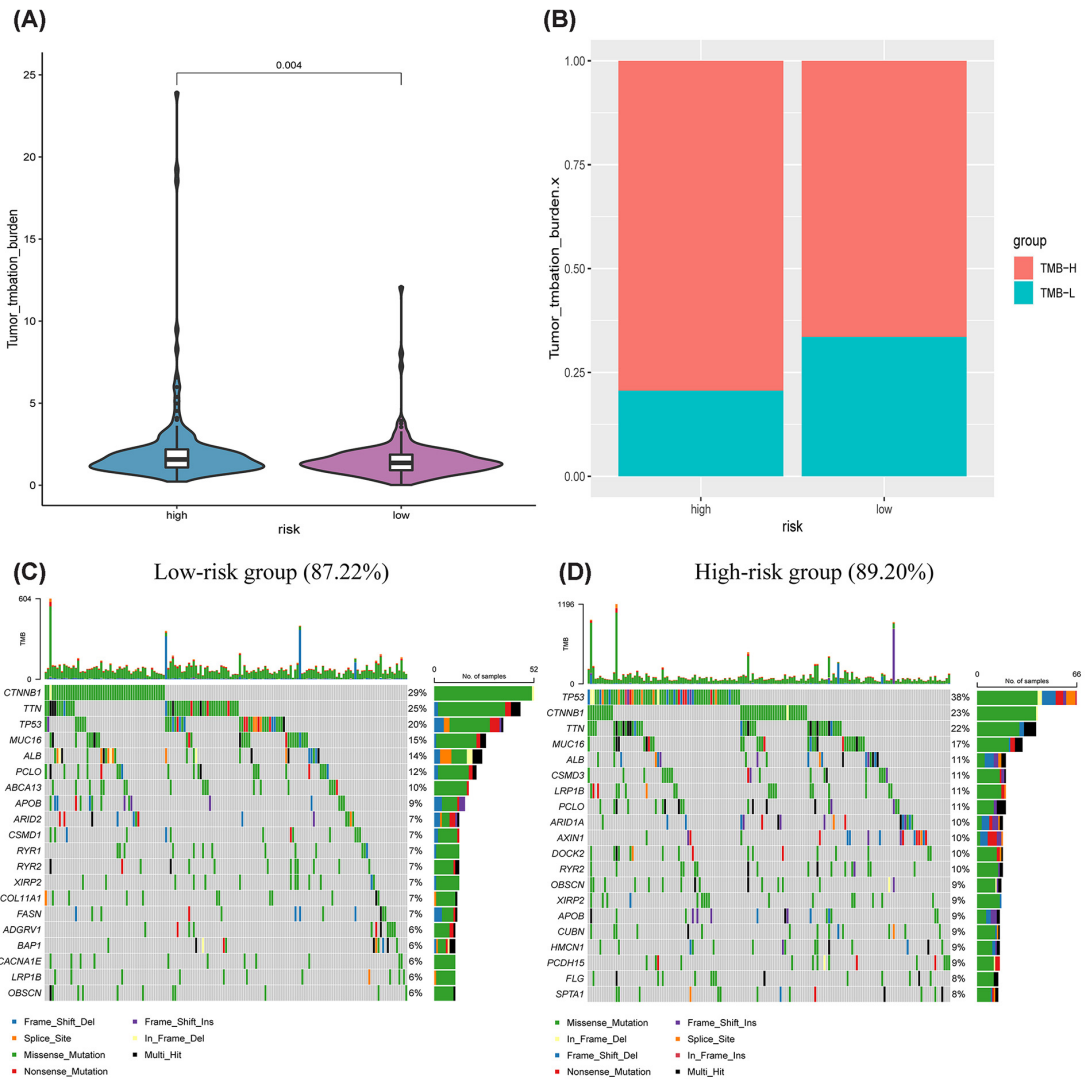
Tumor mutational burden analysis (**A**) Violin plot illustrated a significant difference in tumor mutation burden between high- and low-risk groups (*P*=0.004). (**B**) Percentage bar graph demonstrated the tumor mutation burden in two risk groups. SNP waterfall plots presented mutation information of genes with high frequency mutations in (**C**) low-risk group and (**D**) high-risk group.

The violin plot illustrates a salient variance in TMB across high- and low-risk patients with a *P-*value = 0.004 (Figure 7A). The presence of a higher tumor mutational burden in the high-risk group was further confirmed by the TMB percentage bar graph (Figure 7B). In the intuitive demonstration of SNPs, 87.22% of patients in the low-risk group had mutations (Figure 7C), and 89.2% of patients in the high-risk group had mutations (Figure 7D). The top five mutant genes in the low-risk group were CTNNB1, TTN, TP53, MUC16, and ALB, and the top five mutant genes in the high-risk group were TP53, CTNNB1, TTN, MUC16, and ALB.

### Guidance on immune targets and chemotherapy

Among the 35 immune checkpoints with differences, we detected high expression of immune checkpoints (e.g., PD1 and CTLA4) in the high-risk group (Supplementary Figure S6K), implying that it may be possible that the high-risk group may benefit more from immune-associated therapies against these checkpoints.

By comparing the IC50 of anticancer drugs across high- and low-risk patients, we yielded an aggregate table of 91 drugs that could be used in clinical treatment (Supplementary Table S7). High-risk patients were more sensitive to drugs related to mitosis, the cytoskeleton, protein stability destruction and part of the cell cycle (such as MK-1775, AZD7762, and palbiclib), while low-risk patients were more sensitive to DNA replication-related drugs (e.g., nelarabine, oxaliplatin, fludarabine, and mitoxantrone). The remaining 107 drugs with no difference between the high- and low-risk groups are displayed in Supplementary Table S8, indicating that the model cannot determine the group preference of these drugs. A summary of the effective drugs and their differences is shown in Supplementary Table S9.

### Validation of the prognostic signature

**Figure 8:**
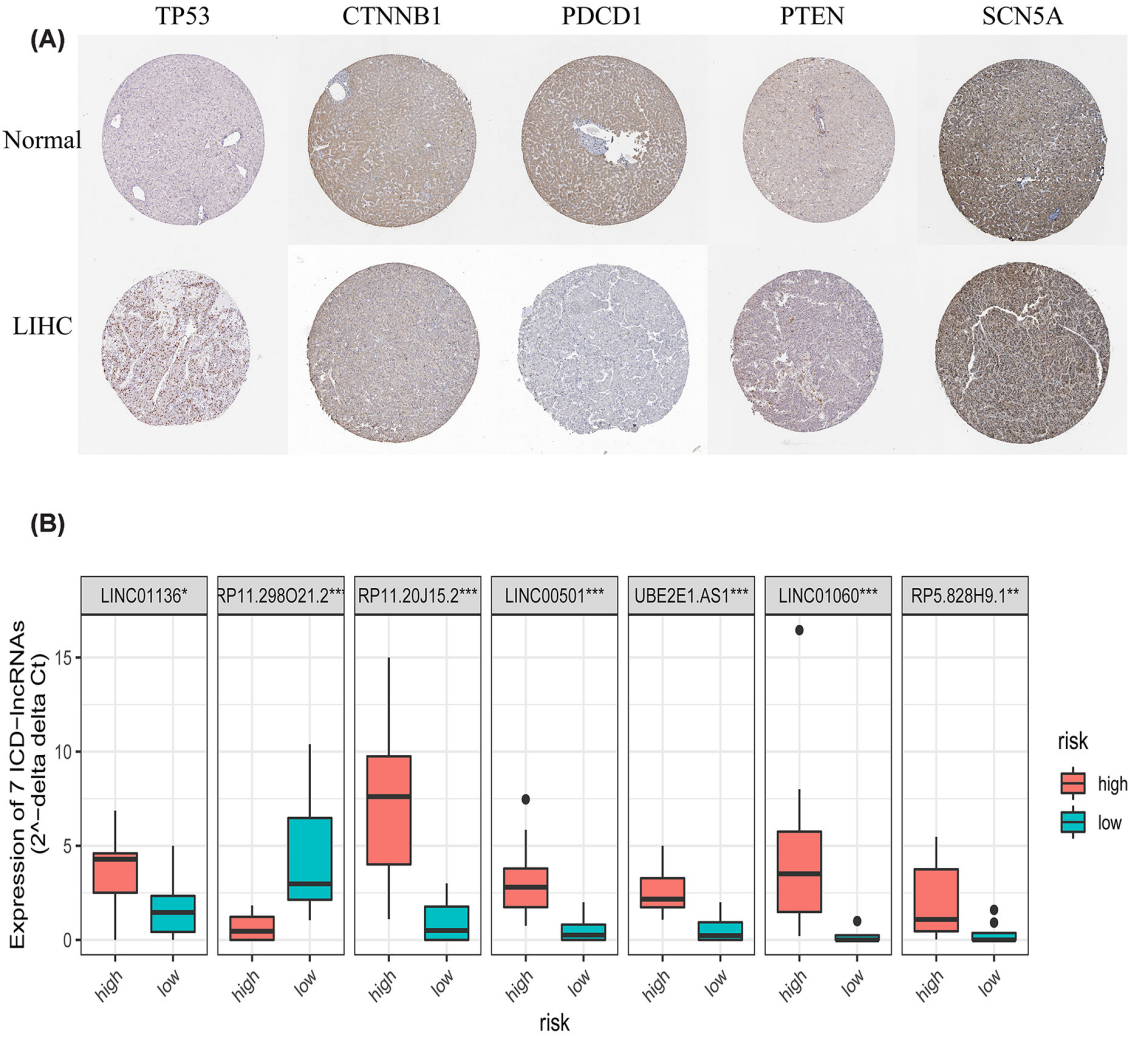
Validation of the ICD prognostic signature (**A**) Immunohistochemical maps of five sets of ICD-associated genes (TP53, CTNNB1, PDCD1, PTEN, and SCN5A) from the HPA database. (**B**) RT-qPCR results of differential expression of seven risk ICD lncRNAs in two segments. *P<0.05, **P<0.01, ***P<0.001.

We derived the protein expression levels of five of the ICD-associated genes in HCC and normal tissues from the HPA database (Figure 8A).

The expression of seven ICD-associated risk lncRNAs was validated in RT-qPCR experiments in high- and low-risk patients. The results demonstrated that all seven lncRNAs were differentially expressed (Figure 8B), and all but RP11.298O21.2 were highly expressed in the high-risk group.

## Discussion

HCC is a global health concern, with the incidence reaching >90,000 cases by 2025 [25,26]. ICD is a modality that is closely associated with tumorigenesis development by recognizing surface antigens that further induce immune cell collections on tumor cells, thereby promoting necrosis through immune cell infiltration [27]. To address the prediction problem, we established a prognostic signature of LINC01136, RP11.298O21.2, RP11.20J15.2, LINC00501, UBE2E1.AS1, LINC01060, and RP5.828H9.1 in HCC and plotted a nomogram to assess patient prognosis. The results of the survival analysis showed that our signature has good assessment ability, and the calibration curve indicated that the risk score added predictive accuracy to the nomogram.

Previous studies have shown that ICD is closely related to tumor development [27]. Hence, many researchers have experimented with ICD-lncRNAs to build prognostic signatures. In the ICD-lncRNA model of HNSC, patients in the high-risk group presented a noticeably dismal prognosis (*P*<0.0001 in TCGA, *P*=0.013 in GSE41613) [9]. The same results were demonstrated in ICD-lncRNA models of UM (*P*<0.01 in TCGA, *P*=0.009 in GEO) and GBMLGG (*P*<0.001 in TCGA, *P*=0.003 in CGGA) [10,11]. Consistent with the prognostic signature constructed in the present study (*P*<0.001 in the training cohort, *P*=0.029 in the testing cohort), the prognosis was worse in the high-risk group. In these studies, we noticed that immune recognition sites and killer immune cells were less abundant in the high-risk group. This led to the assumption that ICD was an important process of self-protection against tumors. Collectively, the ICD-associated prognostic signature was a worthy method for evaluating prognosis.

The progression of HCC is strongly related to the dysregulation of the host's immune mechanisms. Alessandro Granito et al. described that Tregs, with CD4+ CD25+ Foxp3+ as molecular markers, had a suppressive effect on CD8+ T cells and NK cells in the development of HCC, thus reducing the effect of the host immune system on tumor cell death [28]. Consistent with the results of the present study, ssGSEA results revealed a significant elevation of Tregs in the high-risk group, accompanied by a decrease in NK cells. Consequently, we speculated that the detrimental effect of ICD on tumor cells in the high-risk group was suppressed by Tregs so that the effect of the immune system on HCC cell death was reduced, leaving the patients in the high-risk group without the protection of ICD and causing a poor prognosis.

The GSEA results indicated that the malignancy of HCC in the high-risk group was related not only to the TME but also to HCC cells. Lu’s study illustrated that c-MYC and cyclin D1 were both targets of the WNT pathway, and up-regulation of β-catenin produced by the WNT pathway led to up-regulation of both targets [29]. In addition, Fubo Ji et al. found that significant up-regulation of P450 could hinder transcription of the downstream gene c-MYC and prevent HCC [30], consistent with our analysis that P450 was up-regulated in the low-risk group and c-MYC was enriched in the high-risk group. Hence, the combined effect of enhancing P450 expression and inhibiting c-MYC expression can be considered to enhance the prognosis of patients.

The results of the drug sensitivity prediction analysis provided guidance for individualized treatment after evaluation. Immunotherapy and chemotherapeutic agents are two of the commonly used options for HCC [31]. Sagar et al. found that ICD could be mediated through the p38 pathway, leading to the effective death of tumor cells [32]. Zhuo et al. showed that directing apoptosis can effectively increase ICD and thus achieve anti-HCC effects [5]. EGFR signaling drugs can inhibit cell proliferation and enhance ICD by increasing the permeation of CD8+ and NK cells for the treatment of HCC [33]. Furthermore, Bernardo Stefanini et al. found that the combination of TKIs with ICIs is expected to be a mainstream regimen in the future [34]. While reducing immunosuppression through immune targeting and strengthening ICD, this combination also suppressed tumor proliferation by inhibiting tyrosine kinases (including EGFR signaling and RTK signaling) and effectively reduced drug resistance during the treatment process. Our drug sensitivity prediction results confirmed that the high-risk group was more sensitive to TKIs than the low-risk group, and the immune checkpoint analysis showed high expression of some popular ICI targets (e.g., PDCD1 and CTLA4) in the high-risk group, which may provide a new direction for personalized treatment of patients in the high-risk group.

Our study established a novel prognostic signature of ICD in combination with lncRNAs in HCC. In the present study cohort, the predictive performance of TMN staging was inferior to that of the obtained prognostic model. Wu et al. established a prognostic signature for ECM-related lncRNA HCC with AUCs of 0.746, 0.683, and 0.670 for time-ROC curves at 1, 3, and 5 years in their TCGA cohort [35]. In a second investigation, Huang et al. developed a genome instability-derived lncRNA-based prognostic signature for HCC with an AUC of 0.619 in a multi-ROC analysis [36]. In the present study, the TCGA cohort time-ROC AUCs of 0.705, 0.691, and 0.689 were significantly higher than those of the proptosis-related prognostic signature, and the AUC of 0.705 for the entire cohort multi-ROC analysis was also better than that of the inflammation-related prognostic signature. We have for the first time aggregated the pathway mechanisms and antitumor drug sensitivities of different risk groups to visualize the potential mechanisms of predictive models and the value of clinical drug guidance. However, it should be acknowledged that our experiments still have shortcomings. First, we had a limited sample size to download from TCGA, and the number of patients with N1-3 and M1 stages was small and all in the high-risk group, so we were unable to perform subgroup survival analysis for N and M staging. Second, we could not obtain BALC staging of HCC patients directly from the TCGA database, so we were unable to compare its predictive performance with that of the model. Finally, we utilized an online database, and subsequent experiments need to be conducted. New data still need to be collected to study the mechanism of these seven ICD-associated lncRNAs.

## Conclusion

We constructed an effective prognostic signature for patients with HCC using seven ICD-lncRNAs, which provided guidance for the prognostic assessment and personalized treatment of patients. In addition, ICD was integrated with WNT pathway-induced immune escape and malignant tumor biological behavior for the analysis of potential mechanisms. To this end, we also provide guidance on clinical agents (e.g., mitogenic inhibitors, JNK, and p38 pathway agents) for the treatment of patients after evaluation. Due to the limitations of experiments and specimens, we need more basic, mechanistic and clinical studies for validation.

## Supplementary Material

Supplementary Figures S1-S6 and Tables S1-S9

## Data Availability

The data sets used and/or analyzed during the current study are available from the corresponding author upon reasonable request.

## Abbreviations

95%H: 95% high
95%L: 95% low
APC: antigen-presenting cell
AUC: area under curve
C-index: Concordance index
CGGA: Chinese Glioma Genome Atlas
Coef: coefficient
DEL: differential expression of long non-coding ribonucleic acid
FDR: false discovery rate
GEO: Gene Expression Omnibus database
GSEA: gene set enrichment analysis
HCC: hepatocellular carcinoma
HPA database: The Human Protein Atlas database
HR: hazard ratio
IC50: half maximal inhibitory concentration
ICD: immunogenic cell death
ICD-associated gene: immunogenic cell death-related gene
ICD-lncRNA gene: immunogenic cell death long non-coding ribonucleic acid gene
iDAMP: inhibitory damage-associated molecular pattern
IQR: interquartile range
K-M survival analysis: Kaplan–Meier survival analysis
LncRNA: long non-coding ribonucleic acid
M: metastasis
N: node
NES: standardized enrichment score
NK cell: natural killer cell
PCA: principal component analysis
Pr>|*z*|: Probability>|*z* score|
Risk-ICD-lncRNA gene: risk immunogenic cell death long non-coding ribonucleic acid gene
ROC: receiver operating character curve
RT-qPCR: reverse transcription quantitative-polymerase chain reaction
Se(coef): standard error (coefficient)
SNP: single-nucleotide polymorphisms
ssGSEA: single sample gene set enrichment analysis
T: Tumor
TCGA: The Cancer Genome Atlas
Tfh: follicular regulatory T lymphocytes
Th2: helper T 2 cells
Time-ROC: time-dependent receiver operating character curve
TMB: tumor mutation burden
TMN: tumor node metastasis classification
Treg: regulatory T lymphocytes
Z: *Z*-score, standard deviation
