# Supplementary material for: Prognostic value and immune landscapes of immunogenic cell death-related lncRNAs in hepatocellular carcinoma
Source: Biosci Rep. 2023 Sep 5;43(9):BSR20230634. doi: 10.1042/BSR20230634 (PMC10500227; doi:10.1042/BSR20230634)
Supplement: Supplementary Figures S1-S6 and Tables S1-S9 [file BSR-2023-0634_supp.pdf]

Supplementary materials

Figure S1 Heatmap of 7 lncRNA expression and scatterplots of risk score and survival status. (A) Heatmap of 7 lncRNA expression. Riskscore plots for (B) training cohort, (D) testing cohort, (F) and entire cohort. Survival scatterplots for (C) training cohort, (E) testing cohort, (G) and entire cohort.

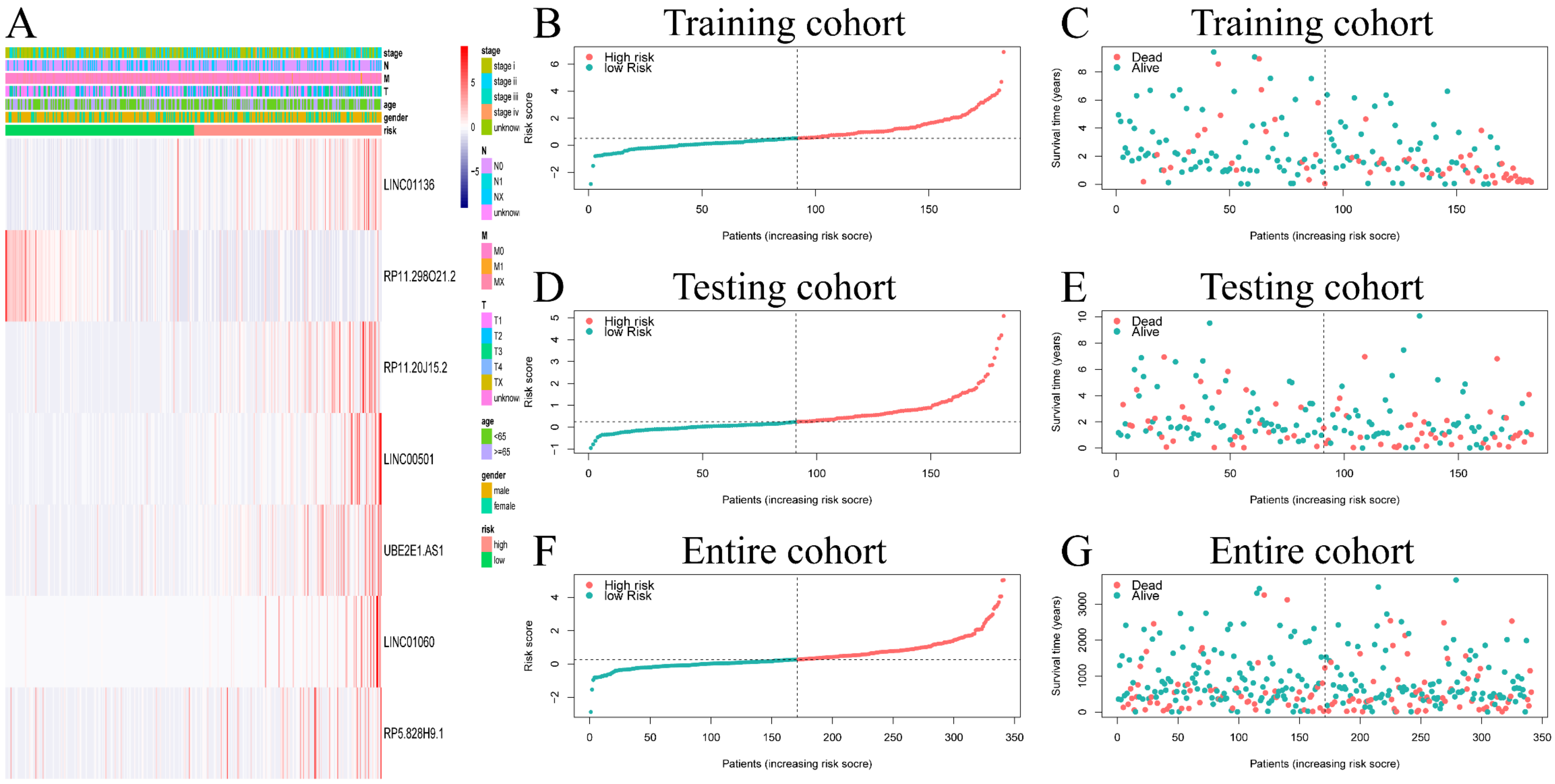

Figure S2 Survival analysis for (A) age <65, (B) age>-=65, (C) female, (D) male, (E) stage I-II, (F) stage II-IV, (G) T1-2, (H) T3-4.

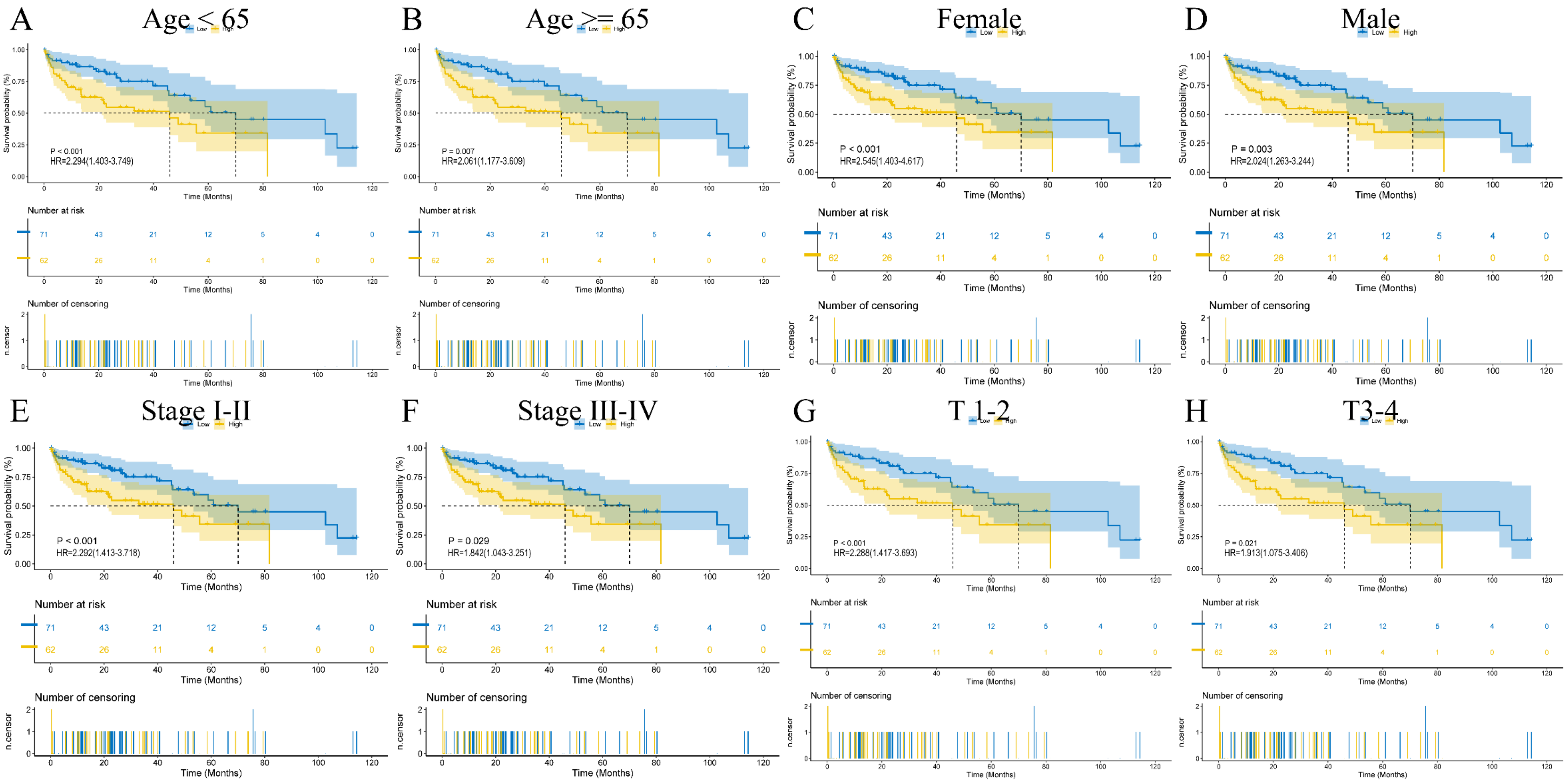

Figure S3 PCA based on (A) all genes, (B) ICD-associated genes, (C) ICD-associated lncRNAs, (D) risk-ICD-associated lncRNAs.

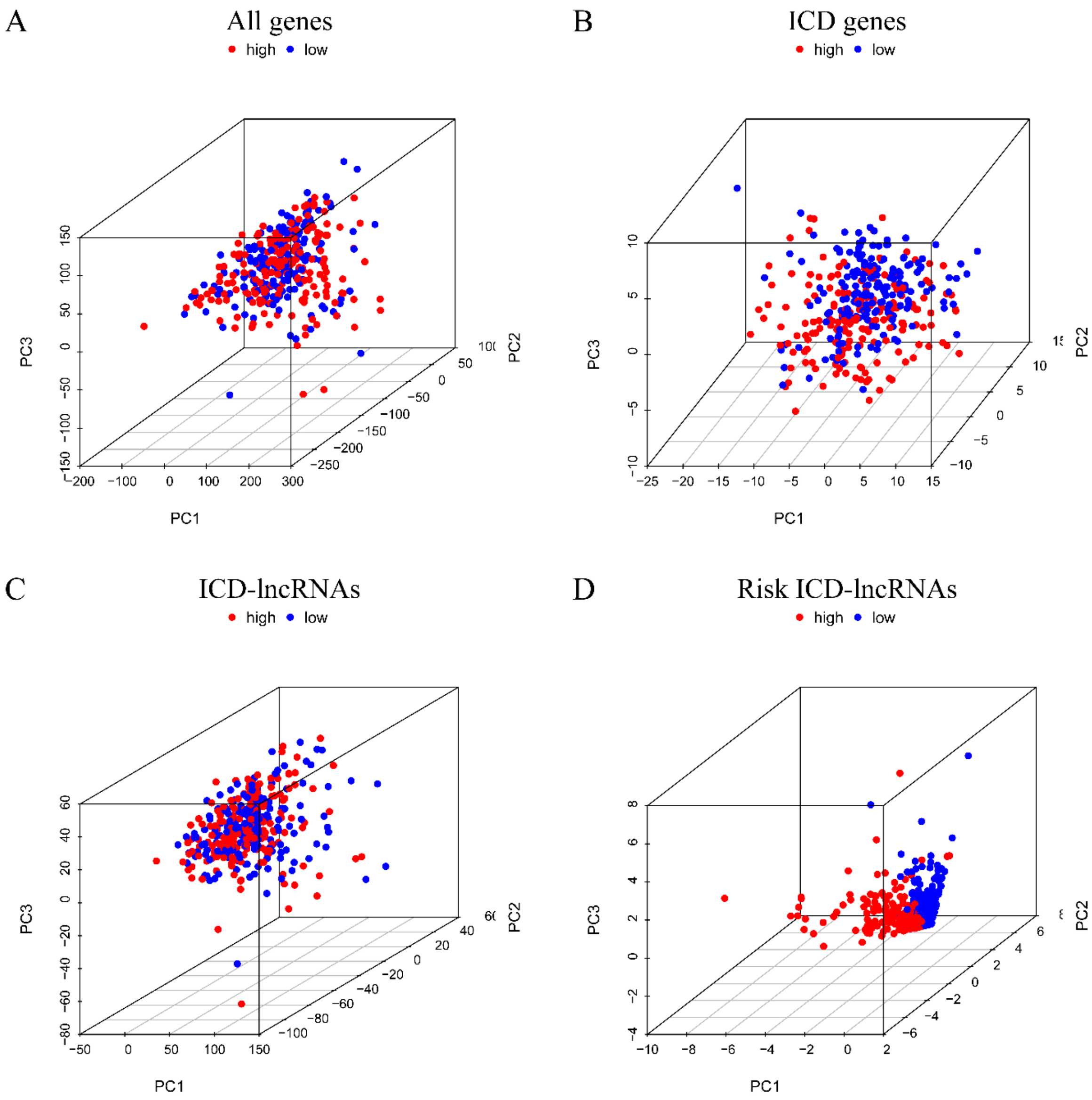

Figure S4 Verification of nomogram accuracy.

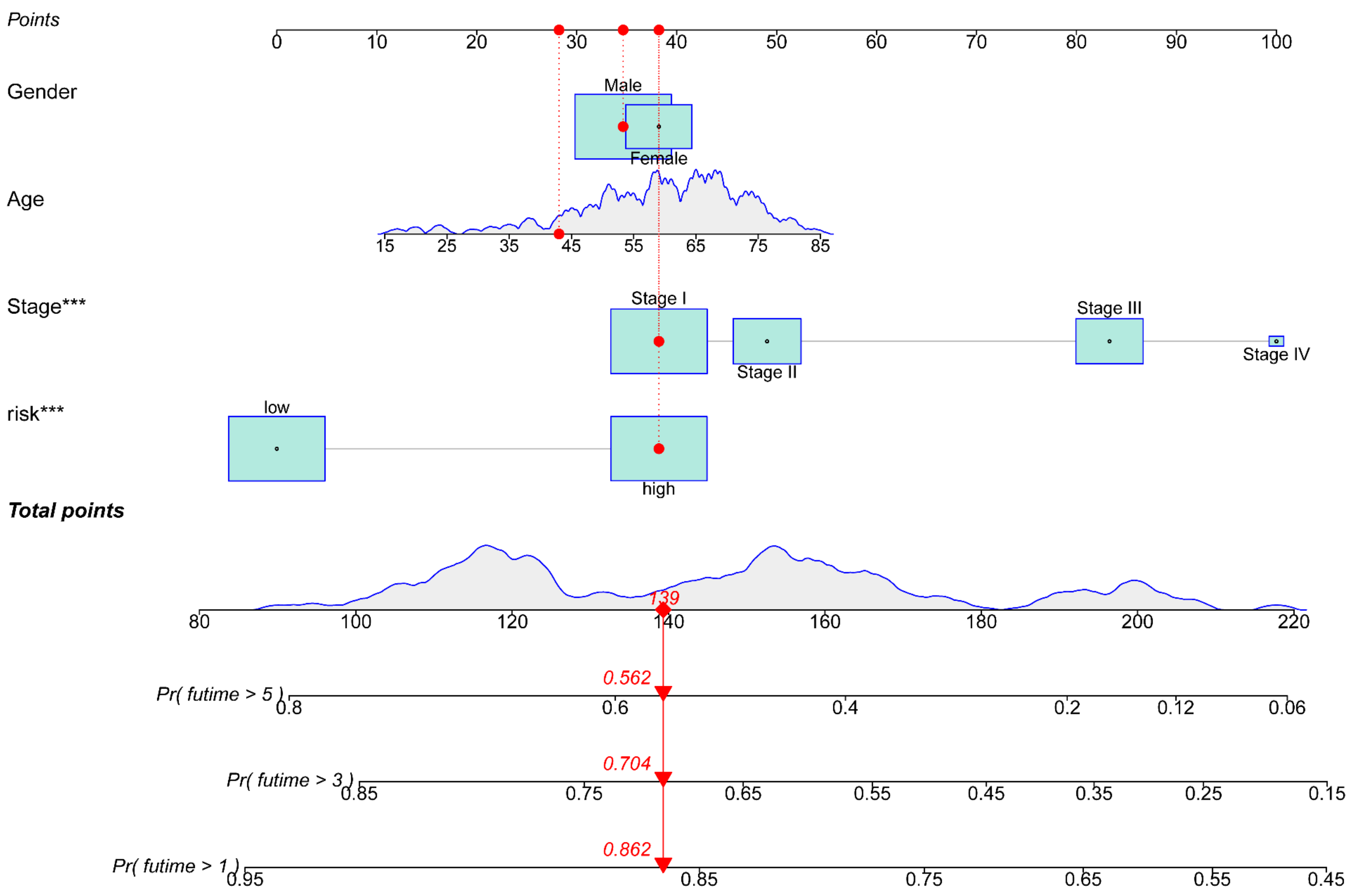

Figure S5 Immune infiltration analysis. (A) Immune cell composition, (B) Immune function analysis, (C) Immune infiltration analysis using 7 different algorithms.

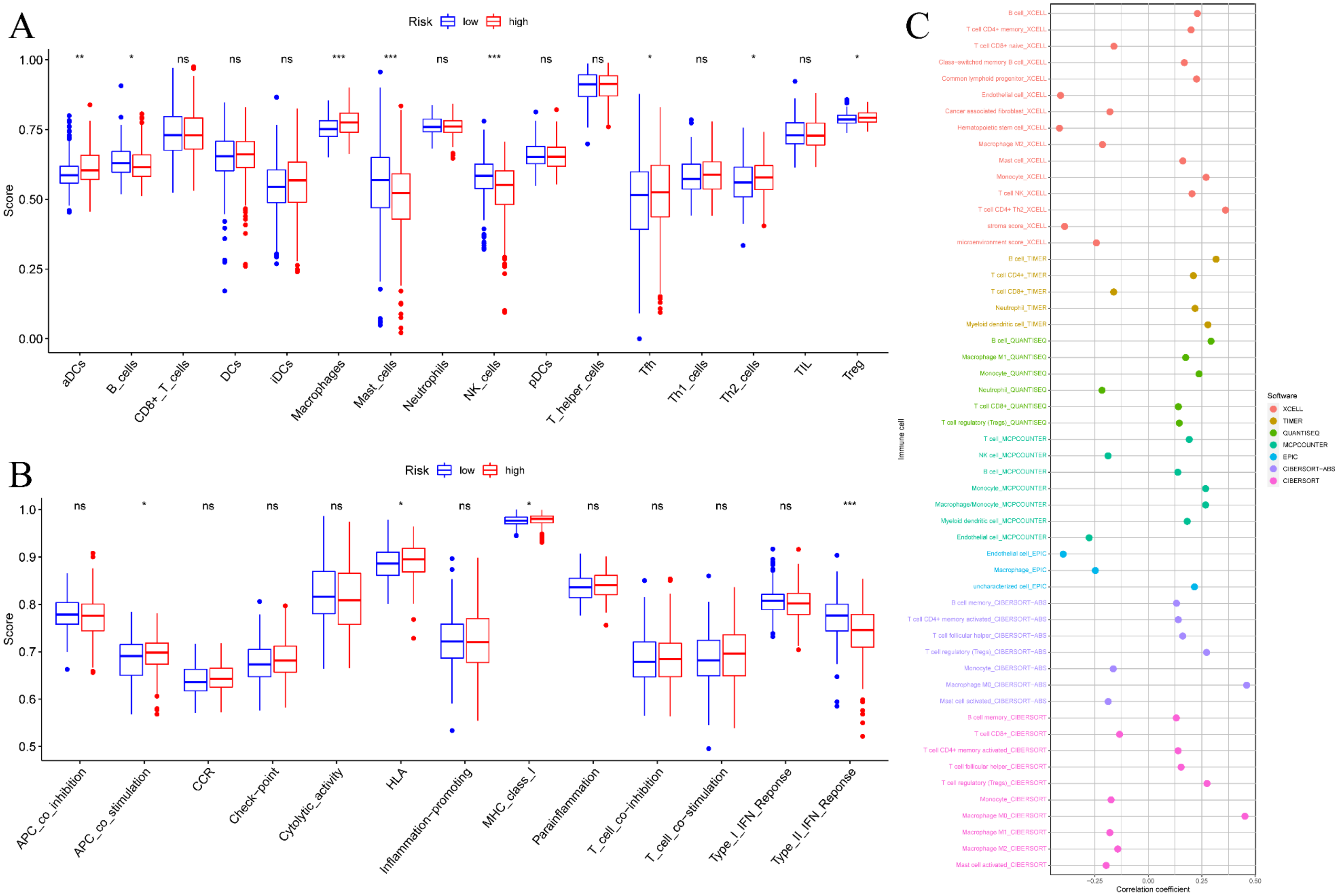

Figure S6 Immune microenvironment relevance and immune checkpoint analysis. (A-J) Scatterplots of immune cell and riskscore correlations, (K) Immune checkpoint expression in two risk segments.

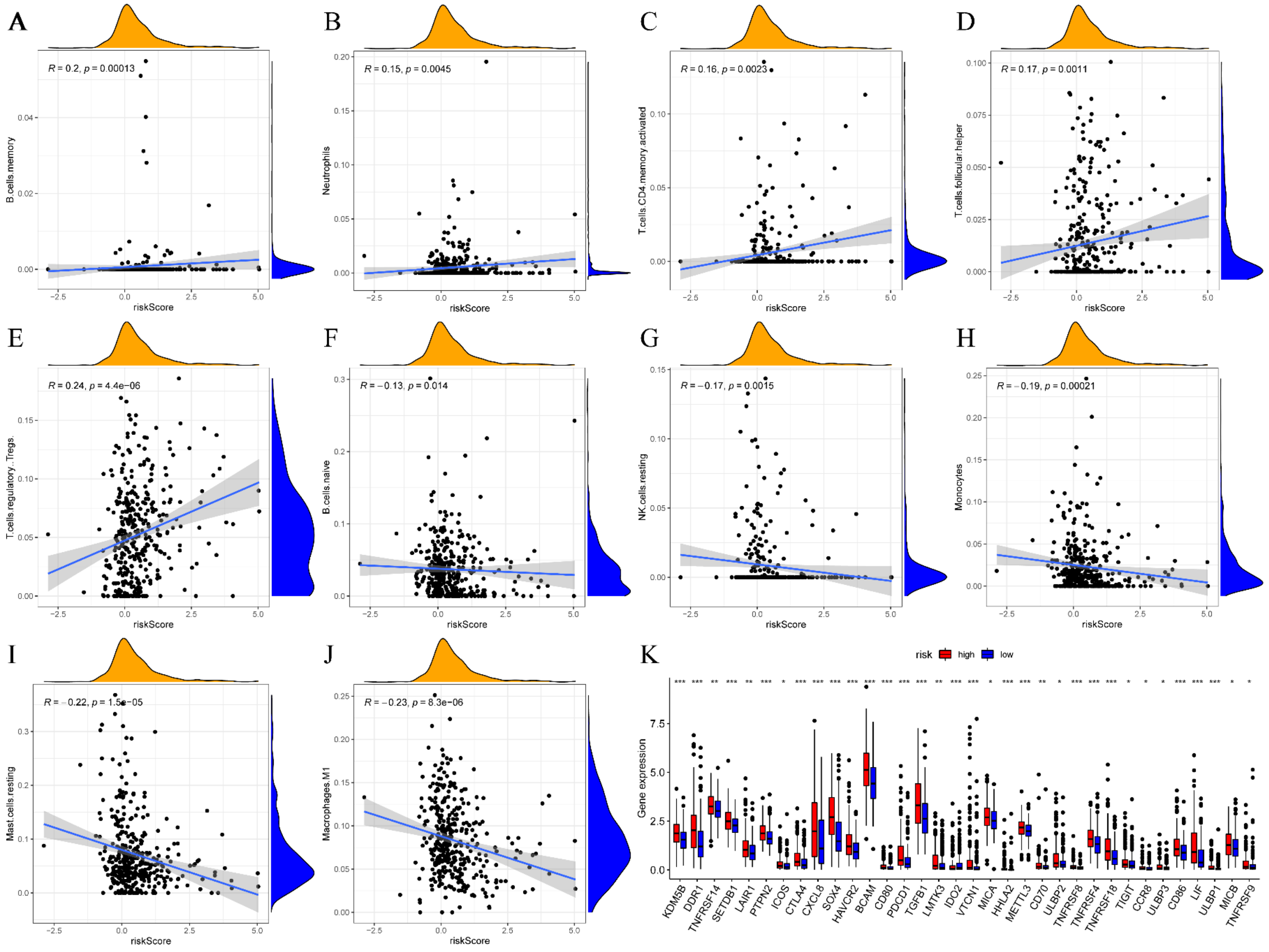

Table S1 Primers for RT-qPCR experiments with 7 ICD lncRNAs.

Gene id	Forward primer	Reverse primer
LINC01136	ACACCCAGTGGCCAAAAGAA	AGAAGAAATCCAGGGGCTGC
RP11-298O21.2	GCACTGTGCTTCCTGCAATC	GCAGCCCTTGGGAAGAAAGAG
RP11-20J15.2	TCCCTCTCGATGCTGTCAGA	AGAGCCAGCATCATTGTCCC
LINC00501	TCTCCTCTCCTGCGGATGAA	CTGTCTTTGGGGGGATGGGGTC
UBE2E1.AS1	ACCCAGCCGAACTCCTTCTA	CTGGAAGCAGGAGGCTGAAA
LINC01060	CCCGTGTCCTTCCCAACATT	TTTGGAGGTGAGGTGGGGTA
RP5-828H9.1	CAAAGAGAGCAGGGAAGGGG	AGGATGTGGCAAAGATGGGG

Abbreviation: RT-qPCR: Reverse transcription quantitative-polymerase chain reaction; ICD: Immunogenic cell death; lncRNA: Long non-coding ribonucleic acid.

Table S2 Results of univariate regression analysis for 243 genes.

Gene	HR	HR (95%L)	HR (95%H)	Gene	HR	HR (95%L)	HR (95%H)
RP11-495P10.8	1.16	1.02	1.33	HDAC11-AS1	6.20	1.01	38.22
RP11-328N19.1	2.01	1.28	3.17	AC004221.2	6.26	1.34	29.31
RP11-92C4.6	0.21	0.06	0.75	AC114812.8	19.44	1.91	197.97
HTR2A-AS1	0.00	0.00	0.50	ELDR	7455.46	4.24	13117033.92
AC004538.3	0.10	0.01	0.86	CTD-2384A14.1	2.96	1.11	7.89
RP11-739N20.2	1.59	1.08	2.33	RP11-78L16.1	1.79	1.08	2.97
RP11-1055B8.9	1.61	1.07	2.43	RP5-1096J16.1	1.86	1.31	2.63
RP11-383J24.1	1.51	1.15	1.99	RP11-332J15.2	1.87	1.39	2.52
LINC01532	2.46	1.34	4.51	LINC00491	2.15	1.06	4.35
RP5-1011O1.2	1.28	1.00	1.63	CTD-2314B22.1	14.11	5.22	38.16
DDX11-AS1	8.86	3.07	25.58	AC073342.12	241.29	1.35	43269.97
KB-1460A1.1	1.65	1.16	2.33	CTD-3035K23.6	4.07	1.81	9.18
CTD-2529O21.1	2.08	1.11	3.90	RP11-510M2.2	2085.29	9.05	480603.17
RP11-445P17.8	1.78	1.12	2.85	CSTF3-AS1	12108.47	31.48	4657271.73
CTD-2116N20.1	2.36	1.23	4.51	RP5-1186N24.3	304.48	7.31	12688.25
PRR7-AS1	3.15	1.02	9.76	CTD-2535I10.1	2.18	1.37	3.46
RP11-336K24.12	3.98	1.09	14.52	AC005281.1	11.61	1.25	108.06
CTD-2199O4.6	3.22	1.46	7.09	RP11-184A2.3	3.99	1.44	11.07
LINC01116	1.36	1.00	1.85	AC006946.16	4.87	1.03	23.06
RP11-676J12.7	0.51	0.28	0.93	RP4-555D20.2	15.94	1.00	253.89
RP11-382D12.2	2.84	1.24	6.49	AC025016.1	2.40	1.62	3.56
RP11-533E19.7	2.25	1.06	4.78	RP11-485F13.1	1.90	1.15	3.14
RP11-608O21.1	2.17	1.21	3.87	RP11-363E6.3	4.47	1.18	16.92
RP11-190C22.8	3.13	1.33	7.38	LINC01012	40.85	1.89	884.05
LINC01136	5.73	2.22	14.79	CTA-796E4.5	74395.76	27.55	200873394.12
ZNF252P-AS1	9.40	1.31	67.43	RP11-47P18.1	1.75	1.10	2.78
LINC01224	3.62	2.12	6.18	RP5-1116H23.4	3.70	1.14	11.95
RP5-1158E12.3	2.99	1.05	8.50	RP11-416N4.1	8.79	2.64	29.24
RP11-108L7.15	4.40	1.95	9.92	RP11-46A10.2	129.90	6.50	2595.96
RP11-363N22.3	32.53	1.93	547.09	CECR7	2.16	1.36	3.43
RP11-261P13.6	3.59	1.00	12.87	RP11-94P11.4	2.97	1.27	6.91
RP11-285J16.1	4.81	1.95	11.87	LINC00668	3.16	1.61	6.19
RP11-277B15.3	2.38	1.02	5.57	LINC00648	1.55	1.05	2.31
AC004160.4	0.28	0.08	0.92	RP11-669N7.2	1.31	1.05	1.64
NOVA1-AS1	2.50	1.40	4.45	RP11-57A19.2	8.97	2.91	27.72
RP11-15E18.1	4.28	1.61	11.37	RP11-843B15.4	5.12	1.32	19.81
KCNMB2-AS1	1.44	1.06	1.95	RP11-325L7.1	1932.34	1.04	3589704.48
LINC01194	2.04	1.31	3.20	RP11-1070N10.5	5.35	1.85	15.51
FIRRE	7.47	2.71	20.58	CTA-397H3.3	20.19	1.51	269.51
RP11-421M1.8	5.60	1.05	29.75	RP11-496D24.2	25.81	2.81	236.79
ATP2A1-AS1	1.82	1.10	2.99	RP4-545L17.12	9.46	2.64	33.96
CTD-2523D13.2	2.89	1.49	5.61	RP11-15B17.1	169.81	1.23	23452.74
LINC01287	1.16	1.00	1.33	RP11-202K23.1	1.58	1.09	2.31
CELSR3-AS1	1.55	1.05	2.29	RP11-314M24.1	1.55	1.01	2.39
RP11-187E13.1	2.53	1.53	4.19	RP11-814H16.2	11.79	1.50	92.43

AC092667.2	3.01	1.33	6.78	CTC-338M12.9	2.26	1.10	4.62
RP1-16A9.1	57.33	2.96	1109.80	CASC11	468157065.11	19806.45	11065637494415.80
RP11-446J8.1	2.28	1.01	5.13	RP11-62J1.4	16.94	1.12	256.59
CDIPT-AS1	4.02	1.73	9.34	RP11-114M1.1	2.96	1.39	6.31
RP11-494H4.3	5.87	1.47	23.49	CTB-138E5.1	4.37	1.93	9.90
DLX6-AS1	6.74	1.18	38.61	RP11-120K24.5	1.80	1.02	3.15
RP11-474G23.3	4.27	1.40	13.06	CH507-154B10.2	436.62	2.61	73123.22
RP11-439C15.4	0.22	0.06	0.85	RP11-519G16.5	1.78	1.09	2.91
RP11-495P10.3	42.08	2.92	606.98	LINC00858	36.91	6.59	206.66
LINC01269	1.52	1.10	2.10	CTD-2619J13.13	6.40	2.63	15.61
CTD-2033A16.3	1.89	1.11	3.23	CTB-35F21.1	25.87	3.86	173.27
LINC01424	29.27	4.19	204.32	RP11-136L23.2	6.81	1.11	41.69
RP11-818F20.5	5.47	1.89	15.77	RP11-1038A11.3	2.14	1.21	3.78
RP11-161I6.2	8.49	3.13	23.04	RP11-78F17.1	21.72	2.30	204.69
LINC00221	1.40	1.11	1.76	RP11-159H10.3	56.41	7.35	432.99
RP11-14N7.2	2.11	1.30	3.43	RP11-169F17.1	1.47	1.02	2.10
LINC00942	1.26	1.07	1.50	CTD-2008P7.8	1.71	1.26	2.32
RP1-118J21.25	6.12	1.27	29.39	RP11-259P20.1	0.00	0.00	0.32
RP11-79O8.1	10.41	1.76	61.47	ERVMER61-1	2.25	1.15	4.41
CTD-2374C24.1	3.13	1.32	7.39	MYHAS	78.31	1.40	4384.79
LINC01117	3.18	1.29	7.79	CTD-2616J11.2	745.00	3.85	143990.65
LRP4-AS1	9.75	1.00	94.90	UBE2E1-AS1	3175.41	84.43	119434.40
RP11-973F15.2	2.19	1.24	3.85	AC005540.3	6.66	1.87	23.69
RP11-79N23.1	3.57	1.25	10.23	RP11-65J3.2	391.09	9.09	16832.65
RP11-320N21.1	330.92	1.90	57729.33	CTD-2623N2.11	143.46	5.48	3756.87
KIAA1614-AS1	35.30	2.49	501.33	RP11-128N14.5	2.92	1.25	6.84
RP11-563N4.1	3.28	1.20	8.91	XXbac-BPG294E21.9	80.52	1.84	3530.64
SPATA3-AS1	214.44	31.56	1456.88	CTD-2118P12.1	2.75	1.07	7.08
RP11-111M22.5	30.51	1.63	569.77	LINC00958	1.49	1.08	2.05
RP13-476E20.1	3.90	1.26	12.04	RP11-317N12.1	1.78	1.17	2.70
RP11-972P1.11	2.89	1.06	7.89	RP11-366L20.2	11861322.86	1142.69	123122319038.14
RP11-219B4.7	54.52	1.16	2554.38	RP11-424M22.3	45.11	5.12	397.58
RP11-874J12.4	2.13	1.43	3.17	IL12A-AS1	54.76	1.56	1927.73
RP5-1157M23.2	9.36	3.30	26.59	CTC-458G6.2	2.64	1.26	5.55
CTD-2591A6.2	1.61	1.08	2.40	LINC01060	51.58	9.97	266.74
RP11-308B16.2	1.64	1.08	2.47	UNC5B-AS1	3.63	1.18	11.14
RP11-461O7.1	0.03	0.00	0.67	RP11-459O1.2	1.38	1.02	1.86
AC087294.2	12.97	1.02	165.38	RP5-1057I20.6	6.10	1.04	35.88
RP4-655J12.4	3.11	1.19	8.14	RP11-74C1.4	32.55	2.32	456.19
RP11-466P24.6	1.64	1.06	2.52	AL591893.1	3833173.50	1.83	8017975516452.58
RP11-89K21.1	2.01	1.13	3.56	LINC00521	18.21	1.06	313.39
AC007128.1	8.54	2.63	27.75	RP11-651P23.5	513.14	7.20	36587.19
RP11-298O21.2	0.02	0.00	0.69	LINC01518	1.52	1.05	2.20
LINC01446	2.48	1.42	4.33	AC012462.1	2.29	1.02	5.12
RP11-29H23.4	2.73	1.13	6.61	RP5-828H9.1	370.81	44.84	3066.43
LINC00184	284.30	2.26	35799.50	LINC01257	8.59	1.71	43.23
BBOX1-AS1	1.75	1.26	2.42	RP11-322E11.2	1129.23	34.64	36814.83
RP11-353N14.2	1.91	1.07	3.39	RP4-660H19.1	2.34	1.32	4.13
RP11-368I23.2	2.24	1.06	4.73	RP5-823G15.5	23.70	4.51	124.58

RP11-552D8.1	95.67	1.72	5321.21	AC007131.1	3.25	1.82	5.80
RP11-485G7.5	3.80	1.24	11.64	RP11-689C9.1	3.46	1.16	10.34
RP11-103J17.2	2.22	1.21	4.05	LINC01579	95.21	2.22	4082.55
RP11-900F13.3	3.77	1.39	10.22	RP4-594A5.1	7.13	1.44	35.20
LL22NC03-N64E9.1	79.94	11.06	577.95	RP11-620J15.2	176573.15	13.11	2377906281.22
CASC20	1.95	1.25	3.04	RP11-1136G4.2	8.01	1.64	39.22
RP11-20J15.2	2.77	1.74	4.41	RP11-501C14.5	1.92	1.17	3.17
LINC00664	9.32	1.57	55.45	RP11-320G10.1	1.77	1.24	2.54
RP5-836N17.4	4.84	1.56	15.02	RP11-578B16.1	3.93	1.48	10.42
AP000569.9	4.10	1.03	16.38	KB-1980E6.3	4.66	1.08	20.01
AC004854.4	3.07	1.20	7.81	RP11-179A16.1	48.81	3.70	644.10
RP5-940J5.3	4.57	1.11	18.81	AC092415.1	4.76	1.71	13.26
LINC00501	18.47	6.69	50.97	RP11-678G15.2	124.19	10.22	1509.32
RP11-758M4.4	2.80	1.57	4.98	RP11-266N13.2	1.94	1.13	3.32
LINC01353	2.76	1.33	5.73	RP11-722M1.1	2.37	1.21	4.65
BPESC1	73.15	2.88	1860.75	RP11-146E13.4	8.68	1.20	62.71
RP11-68L18.1	1009.98	1.09	938860.54	LINC00200	3.16	1.39	7.18
WASIR2	7.05	2.47	20.13	RP11-94B19.6	2.25	1.05	4.84
CTD-2256P15.1	21.87	2.79	171.09	LINC01096	5.17	1.60	16.67
CTA-246H3.12	4.32	1.16	16.00	RP11-305B6.3	2.13	1.15	3.92
RP11-408O19.5	12.66	1.21	132.86	RP11-501O2.5	1.91	1.13	3.22
CTC-327F10.4	3.24	1.56	6.74	RP11-120K24.4	11.89	1.09	129.47
AC018890.6	2.46	1.49	4.05	MIR137HG	936.29	7.20	121774.16
RP11-180C1.1	2.09	1.34	3.26	LINC00162	2.00	1.31	3.07
RP11-146F11.5	6.10	1.39	26.86	RP11-549K20.1	1.48	1.04	2.12
RP11-435O5.4	2.81	1.11	7.11	RP11-567N4.3	24.35	4.28	138.42
RP11-640I15.1	3.14	1.23	7.96	KB-1460A1.3	19.92	3.74	105.97
CASC8	3.09	1.31	7.30				

Abbreviation: HR: Hazard Ratio; 95%L: 95% low; 95%H: 95% high.

Table S3 Genetic information after multi-cox regression analysis.

Gene	Coef	HR	Se(coef)	Z	Pr(>|z|)
LINC01136	1.58	4.84	0.60	2.61	0.01
RP11.298O21.2	-3.01	0.05	1.37	-2.21	0.03
RP11.20J15.2	0.91	2.49	0.32	2.86	0.00
LINC00501	1.51	4.51	0.69	2.18	0.03
UBE2E1.AS1	4.27	71.34	1.67	2.55	0.01
LINC01060	4.07	58.36	0.96	4.25	0.00
RP5.828H9.1	3.25	25.73	1.03	3.15	0.00

Abbreviation: Coef: coefficient; HR: Hazard Ratio; Se(coef): Standard error (coefficient) ; Z: Z score, standard deviation; Pr>|z|: Probability>|z score|.

Table S4 Results of independent prognostic analyses.

Id	Univariate Cox regression				Multivariate Cox regression			
	HR	HR (95%L)	HR (95%H)	P-value	HR	HR (95%L)	HR (95%H)	P-value
Age	1.25	0.87	1.81	0.23	1.22	0.84	1.77	0.31
Gender	0.79	0.55	1.15	0.22	0.86	0.59	1.26	0.44
Stage	1.64	1.34	2.01	0.00	1.46	1.18	1.80	0.00
Riskscore	1.81	1.55	2.11	0.00	1.72	1.47	2.02	0.00

Abbreviation: HR: Hazard Ratio; 95%L: 95% low; 95%H: 95% high.

Table S5 Results of GSEA in the high-risk group.

Category	NES	FDR
**Apoptosis**		
BIOCARTA_FAS_PATHWAY	-1.62	0.002
BIOCARTA_TNFR1_PATHWAY	-1.67	0.001
**Cancer-related pathways**		
BIOCARTA_ATRBRCA_PATHWAY	-1.77	0.000
PID_BARD1_PATHWAY	-1.88	0.000
PID_FOXM1_PATHWAY	-2.01	0.000
**Cell cycle**		
BIOCARTA_CELLCYCLE_PATHWAY	-1.73	0.001
BIOCARTA_G2_PATHWAY	-1.75	0.000
GOBP_NEGATIVE_REGULATION_OF_METAPHASE_ANAPHASE_ TRANSITION_OF_CELL_CYCLE	-2.16	0.000
GOBP_NEGATIVE_REGULATION_OF_NUCLEAR_DIVISION	-2.10	0.000
KEGG_CELL_CYCLE	-2.06	0.000
**DNA mismatch reparation**		
PID_ATR_PATHWAY	-2.07	0.000
PID_FANCONI_PATHWAY	-1.82	0.001
**DNA replication**		
BIOCARTA_MCM_PATHWAY	-1.92	0.000
KEGG_DNA_REPLICATION	-2.01	0.000
**Growth signaling pathway**		
PID_MYC_ACTIV_PATHWAY	-1.73	0.004
PID_MYC_PATHWAY	-1.87	0.000
**Infection**		
REACTOME_INFLUENZA_INFECTION	-2.12	0.000
**Mitosis**		
GOBP_ATTACHMENT_OF_SPINDLE_MICROTUBULES_TO_KINETOCHORE	-2.14	0.000
GOBP_MITOTIC_SPINDLE_ORGANIZATION	-2.13	0.000
PID_AURORA_A_PATHWAY	-1.85	0.000
PID_AURORA_B_PATHWAY	-1.94	0.000
REACTOME_MITOTIC_PROMETAPHASE	-2.13	0.000
REACTOME_MITOTIC_SPINDLE_CHECKPOINT	-2.21	0.000
REACTOME_RESOLUTION_OF_SISTER_CHROMATID_COHESION	-2.15	0.000
**PLK1 pathway**		
PID_PLK1_PATHWAY	-2.10	0.000
**Ribosome production and degradation**		
GOCC_PRERIBOSOME	-2.11	0.000

KEGG_RIBOSOME	-2.11	0.000
KEGG_RNA_DEGRADATION	-1.89	0.000
KEGG_SPLICEOSOME	-2.04	0.000
REACTOME_RRNA_PROCESSING	-2.20	0.000
**WNT pathway**		
BIOCARTA_WNT_PATHWAY	-1.51	0.016

Abbreviation: GSEA: Gene set enrichment analysis; NES: Standardized enrichment score; FDR: False discovery rate.

Table S6 Results of GSEA in the low-risk group.

Category	NES	FDR
**Acute myocardial infarction**		
BIOCARTA_AMI_PATHWAY	2.60	0.000
**Amino acid metabolism**		
GOBP_ALPHA_AMINO_ACID_CATABOLIC_PROCESS	3.31	0.000
GOBP_CELLULAR_AMINO_ACID_CATABOLIC_PROCESS	3.41	0.000
KEGG_GLYCINE_SERINE_AND_THREONINE_METABOLISM	3.09	0.000
KEGG_VALINE_LEUCINE_AND_ISOLEUCINE_DEGRADATION	3.27	0.000
**Coagulation**		
BIOCARTA_INTRINSIC_PATHWAY	2.90	0.000
BIOCARTA_COMP_PATHWAY	2.44	0.000
REACTOME_FORMATION_OF_FIBRIN_CLOT_CLOTTING_CASCADE	2.91	0.000
**Cytochrome metabolism**		
KEGG_DRUG_METABOLISM_CYTOCHROME_P450	3.22	0.000
REACTOME_CYTOCHROME_P450_ARRANGED_BY_SUBSTRATE_TYPE	3.08	0.000
REACTOME_PHASE_I_FUNCTIONALIZATION_OF_COMPOUNDS	3.29	0.000
**Drug metablism**		
REACTOME_ASPIRIN_ADME	3.02	0.000
REACTOME_DRUG_ADME	2.86	0.000
**Lipid metabolism**		
BIOCARTA_NUCLEARRS_PATHWAY	2.60	0.000
GOBP_FATTY_ACID_BETA_OXIDATION	3.31	0.000
GOBP_FATTY_ACID_CATABOLIC_PROCESS	3.70	0.000
KEGG_FATTY_ACID_METABOLISM	3.42	0.000
**Metabolism**		
GOBP_MONOCARBOXYLIC_ACID_CATABOLIC_PROCESS	3.38	0.000
GOBP_EPOXYGENASE_P450_PATHWAY	2.90	0.000
**Transcription factor network**		
PID_HNF3A_PATHWAY	1.92	0.005
PID_HNF3B_PATHWAY	1.85	0.006
**Vitamin metabolism**		
KEGG_RETINOL_METABOLISM	3.35	0.000

Abbreviation: GSEA: Gene set enrichment analysis; NES: Standardized enrichment score; FDR: False discovery rate.

Table S7 IC50 of clinical drug sensitivity for HCC patients in high and low risk groups.

Drug	High-risk group		Low-risk group		P-value	Sensitive groups
	Media	IQR	Media	Q1		
**Apoptosis regulation**						
Sepantronium bromide	0.01	0.01-0.02	0.02	0.01-0.03	0.00	high
UMI-77	14.36	9.8-20.31	16.20	12.91-21.35	0.00	high
WEHI-539	34.46	23.56-45.33	38.16	28.1-47.9	0.02	high
Navitoclax	6.39	4.22-9.53	7.47	5.35-10.37	0.03	high
MIM1	48.56	36.99-62.59	53.03	44.21-61.86	0.03	high
LCL161	142.00	119.46-180.6	134.44	107.97-151.52	0.00	low
ABT737	9.52	6.48-13.2	7.96	5.89-11.25	0.02	low
**Cell cycle**						
MK-1775	1.49	0.98-2.37	2.19	1.62-3.04	0.00	high
Wee1 Inhibitor	6.78	4.57-9.75	8.49	6.54-11.74	0.00	high
AZD7762	1.00	0.69-1.53	1.25	0.92-1.74	0.00	high
Palbociclib	36.76	25.86-51.25	42.76	31.38-55.98	0.02	high
Dinaciclib	0.06	0.04-0.08	0.06	0.05-0.08	0.02	high
RO-3306	20.84	19.35-22.49	20.11	19.1-21.55	0.01	low
**Chromatin histone acetylation**						
Vorinostat	4.02	3.22-5.14	4.38	3.79-5.09	0.02	high
OF-1	62.42	51.43-76.63	56.99	48.6-67.54	0.00	low
EPZ5676	265.93	218.53-319.23	246.95	210.34-289.98	0.01	low
**Chromatin other**						
I-BRD9	77.20	58.86-101.91	85.75	67.53-101.97	0.03	high
JQ1	10.99	7.54-16.61	10.00	7.09-13.63	0.05	low
**Cytoskeleton**						
BDP-00009066	10.50	8.22-12.48	11.52	9.13-13.79	0.00	high
PAK_5339	10.96	9.13-12.42	11.53	9.76-13.03	0.04	high
**DNA replication**						
Leflunomide	147.69	122.74-168.01	153.49	134.75-174.22	0.02	high
Nelarabine	437.48	337.25-574.77	396.12	328.17-487.91	0.01	low
Oxaliplatin	44.68	31.3-60.83	38.82	29.16-53.96	0.01	low
Fludarabine	161.65	120.5-223.55	141.94	106-201.6	0.02	low
Mitoxantrone	2.01	1.27-3.31	1.64	0.99-2.82	0.02	low
**EGFR signaling**						
Lapatinib	19.09	14.91-24.95	23.46	18.28-28.22	0.00	high
Sapitinib	52.86	39.94-65.41	57.21	45.48-77.5	0.00	high
Osimertinib	5.69	4-7.55	6.41	5.04-8.07	0.01	high
Afatinib	6.38	4.53-8.11	6.80	5.35-8.65	0.02	high

AZD3759	15.28	12.79-18.55	13.78	11.46-16.95	0.01	low
**ERK MAPK signaling**						
ERK_6604	27.64	20.1-38.58	33.50	26.76-45.69	0.00	high
Ulixertinib	15.93	12.04-20.51	16.40	13.26-21.01	0.00	high
VX-11e	15.30	10.69-21.62	18.48	13.5-26.19	0.00	high
SCH772984	12.19	8.03-18.89	14.87	10.41-20.56	0.00	high
Selumetinib	69.46	47.11-103.13	58.25	40.13-86.53	0.01	low
PLX-4720	91.31	68.27-119.5	78.85	62.04-101.72	0.00	low
**Genome integrity**						
Telomerase Inhibitor IX	1.55	1.07-2.06	1.90	1.49-2.3	0.00	high
AZD6738	7.07	4.71-10.2	8.67	6.64-12.59	0.00	high
VE821	57.98	43.38-80.02	69.22	53.17-83.72	0.00	high
Niraparib	77.85	59.16-105.6	70.17	56.94-93.17	0.03	low
NU7441	14.36	12.65-16.05	12.93	11.68-14.4	0.00	low
KU-55933	81.57	67.59-102.6	72.09	59.85-86.58	0.00	low
**Hormone-related**						
GDC0810	135.55	106.23-158.97	148.75	124.73-167.09	0.00	high
**IGF1R signaling**						
BMS-536924	8.13	6.15-10.24	8.68	7.38-10.67	0.01	high
**JNK and p38 signaling**						
Doramapimod	96.92	86.76-114.69	83.31	74.07-92.96	0.00	low
**Metabolism**						
Daporinad	0.01	0.01-0.02	0.01	0.01-0.02	0.00	both
**Mitosis**						
Paclitaxel	0.05	0.03-0.08	0.09	0.05-0.12	0.00	high
Vinblastine	0.02	0.01-0.03	0.03	0.02-0.04	0.00	high
Vinorelbine	0.04	0.02-0.06	0.05	0.04-0.08	0.00	high
Tozasertib	17.82	13.19-24.05	20.89	16.83-25.1	0.00	high
Vincristine	0.15	0.08-0.25	0.21	0.12-0.34	0.00	high
Docetaxel_1819	0.09	0.05-0.16	0.11	0.07-0.18	0.01	high
Docetaxel_1007	0.01	0.01-0.01	0.01	0.01-0.02	0.00	high
**Other**						
Pevonedistat	1.64	1.06-2.94	2.41	1.6-3.63	0.00	high
BPD-00008900	89.56	72.45-111.07	99.96	83.87-118.18	0.00	high
YK-4-279	8.16	5.2-13.81	11.24	8.03-16.09	0.00	high
IAP_5620	173.24	141.06-239.5	154.51	117.9-204.5	0.00	low
Dactinomycin	0.09	0.06-0.12	0.08	0.06-0.1	0.04	low
Picolinici-acid	177.80	151.09-197.85	164.31	148.67-191.55	0.05	low

LY2109761	185.51	143.48-237.18	162.56	124.62-193.27	0.00	low
**Other, kinases**						
ULK1_4989	9.51	6.06-12.7	12.29	8.55-16.29	0.00	high
Dasatinib	5.25	2.75-8.35	6.43	3.9-10.69	0.00	high
Ibrutinib	86.80	62.83-118.08	99.73	73.87-126.34	0.01	high
AZD1208	210.23	167.82-253.16	179.48	149.58-221.63	0.00	low
JAK1_8709	70.58	54.51-94.54	54.04	43.5-69.54	0.00	low
**p53 pathway**						
Nutlin-3a (-)	117.28	65.78-206.28	91.02	54.92-152.22	0.00	low
**PI3K/MTOR signaling**						
Buparlisib	2.50	2.08-2.91	2.66	2.34-3.12	0.00	high
GNE-317	1.48	1.2-2.1	1.77	1.42-2.26	0.00	high
Pictilisib	3.94	2.94-5.29	4.57	3.42-5.62	0.01	high
Alpelisib	32.98	18.99-54.04	40.28	29.87-53.33	0.01	high
PF-4708671	45.86	39.3-54.61	50.20	43.07-57.71	0.02	high
Rapamycin	0.11	0.08-0.15	0.12	0.1-0.15	0.04	high
AZD8186	28.39	21.56-37.28	25.29	19.37-32.38	0.01	low
AZD2014	8.03	6.17-10.91	7.17	6.01-8.92	0.01	low
LJI308	164.26	135.92-212.89	152.39	124.2-189.96	0.00	low
Uprosertib_1553	22.10	16.22-29.62	18.01	13.91-22.79	0.00	low
Uprosertib_2106	17.67	12.98-23.8	13.57	10.63-19.96	0.00	low
Afuresertib	13.55	9.68-18.58	12.17	9.26-14.94	0.00	low
AZD6482	24.67	18.22-35.27	20.02	15.00-25.84	0.00	low
**Protein stability and degradation**						
ML323	83.31	67.72-95.49	96.87	81.45-112.5	0.00	high
MG-132	0.19	0.16-0.22	0.21	0.19-0.24	0.00	high
Bortezomib	0.01	0.01-0.01	0.01	0.01-0.01	0.00	both
**RTK signaling**						
Staurosporine	0.04	0.03-0.07	0.05	0.04-0.08	0.00	high
Axitinib	21.27	17.82-24.47	22.87	19.93-25.48	0.00	high
Cediranib	8.88	6.23-11.59	9.50	7.61-11.61	0.04	high
SB505124	10.19	9.67-10.81	9.69	9.32-10.26	0.00	low
**WNT signaling**						
WIKI4	39.23	32.85-45.67	42.87	38-47.82	0.00	high
Wnt-C59	65.01	55.7-78.78	71.35	62.95-80.63	0.00	high
XAV939	81.52	72.16-95.18	76.41	68.2-90.32	0.01	low
SB216763	185.97	142.12-241.97	174.62	136.59-212.49	0.03	low

Abbreviation: IC50: half maximal inhibitory concentration; HCC: Hepatocellular carcinoma; IQR: Interquartile range.

Table S8 IC50 of clinical drug sensitivity for HCC patients in high and low risk groups with no difference.

Drug	High-risk group		Low-risk group		P-value
	Media	IQR	Media	IQR	
**ABL signaling**					
Nilotinib	36.85	29.73-46.19	37.93	30.87-46.66	0.28
**Apoptosis regulation**					
Obatoclax Mesylate	4.06	3.35-4.93	4.01	3.4-4.64	0.75
Sabutoclax	0.68	0.52-0.86	0.65	0.55-0.78	0.48
Venetoclax	8.95	7.08-11.23	8.44	7.27-10.3	0.35
AZD5991	82.18	49.77-134.08	70.23	50.79-107.1	0.09
AZD5582	9.15	5.95-14.29	10.84	6.09-15.44	0.08
**Cell cycle**					
BI-2536	1.32	0.97-1.84	1.33	1.01-1.69	0.77
AZD5438	9.1	7.12-11.88	8.92	6.77-11.94	0.52
Ribociclib	44.06	38.51-50.23	43.89	38.21-48.41	0.48
MK-8776	24.86	16.99-35.06	24.78	19.09-34	0.43
CDK9_5038	0.1	0.07-0.14	0.09	0.07-0.13	0.14
CDK9_5576	0.68	0.54-0.87	0.62	0.5-0.85	0.07
**Chromatin histone acetylation**					
PCI-34051	96.1	74.89-113.71	88.9	74.7-107.97	0.15
Entinostat	8.72	7.12-11.61	8.43	6.64-10.92	0.10
GSK343	17.01	14.57-19.22	16.72	14.67-19.19	0.86
EPZ004777	176.28	138.88-217.65	169.57	144.42-208.41	0.61
GSK591	98.61	77.38-117.65	97.85	83.35-118.13	0.55
**Chromatin other**					
PFI3	193.76	168.47-223.47	191.29	170.22-219.42	1.00
RVX-208	118.13	96.65-144.09	117.65	100.38-140.34	0.96
OTX015	11.97	8.82-16.72	12.08	9.45-15.21	0.89
I-BET-762	28.93	22.22-35.27	27.31	22.92-33.56	0.49
AZD5153	5.39	4.29-7.02	5.07	4.02-6.69	0.25
**Cytoskeleton**					
GSK269962A	18.52	16.11-20.63	18.34	16.47-20.5	0.72
**DNA replication**					
Pyridostatin	29.23	23.04-35.87	29.56	24.05-34.41	0.81
Gemcitabine	0.5	0.25-0.94	0.47	0.23-1.17	0.72
Topotecan	1.13	0.68-1.72	0.96	0.66-1.66	0.55
Irinotecan	12.38	8.73-22.08	11.77	7.93-20.93	0.44
Teniposide	1.68	0.94-2.55	1.7	1.06-2.89	0.34
Cyclophosphamide	177.65	140.4-205.31	176.98	159.13-210.8	0.24
Cisplatin	23.11	14.56-41.11	25.79	17.11-43.58	0.21

Epirubicin	0.34	0.2-0.55	0.38	0.26-0.59	0.12
Temozolomide	401.02	308.48-514.38	435.61	337.48-535.64	0.06
Oxaliplatin	44.68	31.3-60.83	38.82	29.16-53.96	0.05
**EGFR signaling**					
Gefitinib	25.28	21.2-30.51	26.53	21.94-30.92	0.30
Erlotinib	13.56	11.6-16.39	13.01	10.95-16.45	0.26
**ERK MAPK signaling**					
ERK_2440	13.63	10.8-18.86	14.19	10.64-19.22	0.93
PD0325901	1.61	1.14-2.35	1.6	1.14-2.38	0.88
Dabrafenib	101.03	72.65-134.48	106.07	77.42-134.32	0.45
KRAS (G12C) Inhibitor-12	82.12	61.29-107.15	78.46	61.87-94.79	0.31
Ulixertinib	15.93	12.04-20.51	16.4	13.26-21.01	0.18
Trametinib	1.71	1-2.57	1.86	1.15-2.9	0.09
**Genome integrity**					
Talazoparib	22.76	15.38-39.09	23.72	16.6-36.57	0.85
BIBR-1532	146.72	119.57-174.2	144.12	126.47-161.04	0.78
Mirin	112.21	88.85-141.56	112.26	92.11-147.74	0.47
VE-822	28.12	20.27-37.96	28.36	23.06-37.36	0.40
Olaparib	75.92	57.81-103.41	69.47	57.52-88.57	0.11
**Hormone-related**					
Tamoxifen	35.83	30.55-40.33	36.09	31.48-40.71	0.51
Fulvestrant_1816	94.69	75.84-110.32	97.91	82.61-110.58	0.06
Fulvestrant_1200	18.25	15.25-22.44	19.03	16.88-22.25	0.06
**IGF1R signaling**					
BMS-754807	2.07	1.63-2.46	2.1	1.72-2.43	0.77
IGF1R_3801	5.2	3.66-7.66	5.17	3.95-7.58	0.69
GSK1904529A	75.85	63.01-88.64	76.71	63.9-91.59	0.52
NVP-ADW742	15.33	11.1-20.05	16.1	12.87-20.04	0.14
Linsitinib	42.42	32.78-51.75	45.18	37.37-51.36	0.08
**Metabolism**					
AGI-5198	105.4	90.59-116.74	104.56	93.74-118.39	0.46
AGI-6780	62.12	53.64-70.13	62.72	55.26-72.42	0.37
GSK2606414	44.08	33.63-54.49	42.77	32.51-52.43	0.32
**Mitosis**					
ZM447439	19.02	16.27-22.54	18.89	16.26-21.52	0.30
Alisertib	6.81	3.95-10.04	7.12	4.94-10.75	0.09
**Other**					
Dactinomycin	0.09	0.06-0.12	0.08	0.06-0.1	0.79
Eg5_9814	0.05	0.03-0.06	0.04	0.03-0.06	0.63
Cytarabine	5.36	3.25-8.2	5.63	3.6-9.44	0.30
Zoledronate	45.16	35.91-53.74	42.77	37.13-50.2	0.28

VSP34_8731	10.46	9.01-12.38	10.99	9.13-12.64	0.21
TAF1_5496	46.76	36.52-61.71	44.37	37.47-52.25	0.12
BMS-345541	26.93	20.86-35.1	29	23.49-37.39	0.06
5-Fluorouracil	104.75	67.1-176.49	125.13	80.8-177.6	0.05
**Other, kinases**					
IRAK4_4710	139.72	118.01-163.11	139.36	122.82-161.95	0.83
Entospletinib	42.7	33.78-51.12	41.53	33.8-49.68	0.58
Ruxolitinib	133.19	97.21-163.17	126.38	103.36-152.78	0.52
AZD5363	19.56	13.92-28.01	19.36	13.64-25.43	0.46
Sorafenib	13.99	11.57-17.22	14.52	12.41-16.99	0.22
PRT062607	25.46	20.3-31.52	26.18	21.58-32.67	0.20
JAK_8517	20.34	14.14-30.55	18.59	14.46-25.63	0.13
GSK2578215A	133.72	114.4-153.75	138.54	121.48-153.93	0.10
AZ960	7.76	5.29-10.46	8.41	6.06-11.27	0.09
**p53 pathway**					
PRIMA-1MET	98.21	64.8-136.58	101.81	79.18-126.03	0.36
MIRA-1	226.95	174.62-294.85	244.44	194.81-280.61	0.30
**PI3K/MTOR signaling**					
Ipatasertib	34.85	23.95-49.74	35.98	25.63-46.95	0.85
MK-2206	21.32	16.82-26.78	21.24	17.04-25.51	0.56
AZD8055	0.82	0.76-0.89	0.83	0.78-0.88	0.54
CZC24832	161.91	136.44-191.22	159.23	131.63-186.2	0.32
OSI-027	123.61	98.38-149.78	115.6	93.14-146.57	0.25
AT13148	38.75	29.43-52.29	35.42	29.3-47.79	0.14
Dactolisib	0.19	0.14-0.26	0.2	0.16-0.26	0.10
Taselisib	8.09	4.27-13.8	8.85	6.48-13.94	0.07
AMG-319	135.88	105.21-171.17	126.83	103.14-150.13	0.06
**Protein stability and degradation**					
Luminespib	0.09	0.06-0.15	0.1	0.07-0.16	0.14
P22077	86.56	58.19-121.9	93.81	71.6-118.72	0.14
**RTK signaling**					
Foretinib	2.64	2.12-3.48	2.6	2.11-3.47	0.85
Crizotinib	25.57	18.51-34	25.79	21.04-30.78	0.82
AZD4547	17.62	13.62-23.61	18.14	14.69-22.3	0.65
PD173074	56.15	37.22-86.79	61.54	44.59-78.25	0.32
Savolitinib	14.25	10.93-17.02	13.55	10.81-15.83	0.14
AZD1332	47.64	36.59-62.34	48.11	37.46-60.1	0.09
AZD1332	47.64	36.59-62.34	48.11	37.46-60.1	0.09
**Unclassified**					
Elephantin	31.53	22.44-47.25	31.62	26.2-40.55	0.74
Sinularin	36.24	28.93-44.7	35.03	29.31-42.36	0.33
Acetalax	146.81	100.48-204.73	139.35	99.61-181.72	0.19
Podophyllotoxin bromide	0.5	0.38-0.67	0.53	0.43-0.68	0.17

Dihydrorotenone	2.45	1.97-3.21	2.4	1.94-2.8	0.11
Gallibiscoquinazole	13.42	11.14-15.88	14.22	12.14-16.11	0.08
**WNT signaling**					
AZ6102	11.23	9.49-13.84	11.19	9.51-13.38	0.83
IWP-2	16.27	13.54-19.25	15.89	14.04-18.24	0.71
MN-64	117.94	95.54-139.34	110.79	91.46-128.74	0.07
LGK974	54.1	45.42-66.46	57.19	48.23-69.73	0.06
Carmustine	450.23	358.31-578.53	446.65	371.34-532.47	0.66

Abbreviation: IC50: Half maximal inhibitory concentration; HCC: Hepatocellular carcinoma; IQR: Interquartile range.

Table S9 Summary of anti-tumor drug categories.

Category	A: Number of high-risk sensitive	B: Number of significant differences	C: Total quantity of drugs	B/C	A/B
ABL signaling	0	0	1	0.00	NULL
Apoptosis regulation	5	7	12	58.33	71.43
Cell cycle	5	6	12	50.00	83.33
Chromatin histone acetylation	1	3	8	37.50	33.33
Chromatin other	1	2	7	28.57	50.00
Cytoskeleton	2	2	3	66.67	100.00
DNA replication	1	5	15	33.33	20.00
EGFR signaling	4	5	7	71.43	80.00
ERK MAPK signaling	4	6	12	50.00	66.67
Genome integrity	3	6	11	54.55	50.00
Hormone-related	1	1	4	25.00	100.00
IGF1R signaling	1	1	6	16.67	100.00
JNK and p38 signaling	0	1	1	100.00	0.00
Metabolism	1	1	4	25.00	100.00
Mitosis	7	7	9	77.78	100.00
Other	3	7	15	46.67	42.86
Other, kinases	3	5	14	35.71	60.00
p53 pathways	0	1	3	33.33	0.00
PI3K/MTOR signaling	6	13	22	59.09	46.15
Protein stability and degradation	3	3	5	60.00	100.00
RTK signaling	3	4	11	36.36	75.00
Unclassified	0	0	6	0.00	NULL
WNT signaling	2	4	9	44.44	50.00
